# Experimental Study on Indirect CO_2_ Mineralization of Industrial Solid Wastes: Electric Arc Furnace (EAF) Slag and Nickel Mine Tailings

**DOI:** 10.1021/acsomega.5c05128

**Published:** 2025-09-04

**Authors:** Hamid Radfarnia, Katrin Staneva, Ahmed Shafeen, Kourosh Zanganeh, Bussaraporn Patarachao, Stephannie Vasquez Huertas, Andre Zborowski, Judy Kung, Seyedeh Laleh Dashtban Kenari, Sanaz Mosadeghsedghi, Konstantin Volchek

**Affiliations:** † Natural Resources Canada, 113679 CanmetENERGY, 1 Haanel Drive, Ottawa, ON K1A 1M1, Canada; ‡ 6356 National Research Council, Energy, Mining and Environment, 1200 Montreal Road, Ottawa K1A 0R6, Canada; § Natural Resources Canada, CanmetMINING, 555 Booth Street, Ottawa, ON K1A 0E9, Canada

## Abstract

CO_2_ mineralization utilizing alkaline industrial wastes as feedstocks is a promising approach for long-term carbon sequestration. This study investigates the indirect carbonation of electric arc furnace (EAF) steel slag and nickel mine tailing using two leaching agents, inorganic hydrochloric acid (HCl) and waste-derived acid mine drainage (AMD). The efficiency of metal leaching and CO_2_ sequestration capacity were evaluated under varying process conditions, including temperature and carbonation reaction acceleration by ultrasound cavitation. Results demonstrated that AMD can be a viable alternative to HCl, potentially reducing chemical costs while aiding in mine waste remediation. However, the effectiveness of both leaching and carbonation processes is strongly influenced by the mineralogical composition of the feedstocks. For serpentine-based feedstock rich in magnesium (nickel tailing), using AMD as a leaching agent resulted in a sequestration capacity of up to 65.12 g-CO_2_ /kg-Ni-Tailing versus 18.28 g-CO_2_ /kg-Ni-Tailing with HCl at room temperature. Conversely, for EAF steel slag rich in calcium, the trend was opposite, with HCl achieving a sequestration capacity of up to 154.76 g-CO_2_/kg-EAF versus 63.58 g-CO_2_/kg-EAF with AMD. The study further explores the impact of operating temperature and ultrasound process intensification on reaction kinetics, concluding that CO_2_ sequestration efficiency was improved either by increasing the temperature or employing ultrasound processing. These findings contribute to the development of sustainable mineralization strategies for industrial waste valorization and greenhouse gas mitigation.

## Graphical abstract

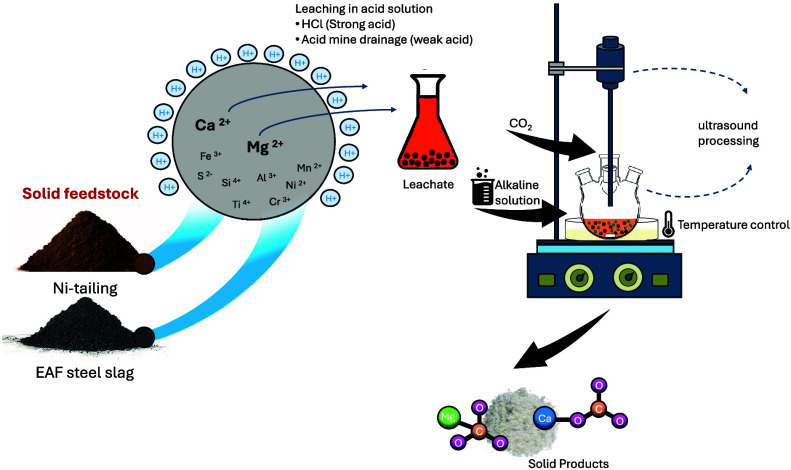

## 1 Introduction

In response to increasing global carbon emissions, there is an urgent need to accelerate the development of efficient and sustainable carbon capture, utilization, and storage (CCUS) technologies. Several storage techniques, such as geological or mineralization sequestration^1,2^ and biological carbon fixation,^3^ have garnered attention due to their promising CO_2_ uptake performances and potential long-term storage capabilities, preventing CO_2_ from being emitted back into the atmosphere.

Among these techniques, the ex-situ CO_2_ mineralization is widely recognized as an environmentally friendly, low-risk method that stores CO_2_ by reacting it with Mg- and Ca-bearing materials to form stable carbonate**s**. Its use of abundant surface-accessible alkaline materials and lower operational risks makes it a promising option for large-scale CO_2_ storage compared to subsurface mineralization.^2,4,5^

Recently, the application of natural or industrial solid waste materials with Ca and/or Mg constituents has received increasing attention. Suitable materials for mineralization include a variety of sources such as serpentine-rich mine wastes or tailings (Mg-bearing), natural serpentine rocks rich in Mg, and Ca-rich industrial byproducts like steel slags and fly ash.^6–8^ A study performed in 2019 estimated that industrial alkaline byproduct or waste materials will have a CO_2_ storage potential of 2.9–8.5 billion tonnes per year by 2100.^9^

Ex-situ carbonation processes typically suffer from low reaction efficiency and high energy consumption for reagent recovery.^2,5,7,10^ To address these challenges, the indirect aqueous CO_2_ carbonation route—based on the pH-swing mechanism—has been extensively studied for improving the performance of mineral carbonation systems.^7,11–14^ In this process, alkaline constituents (Ca^2+^ and/or Mg^2+^) are extracted using acidic or saline solutions, then react with CO_2_ to form stable carbonates.

Among the available raw materials, industrial solid wastes (ISW), namely, electric arc furnace (EAF) steel slags and mining-based materials (e.g., mine tailings) have demonstrated significant potential for use in the indirect aqueous CO_2_ mineralization route.^9,15–20^ Carbonation of mine tailings offers permanent CO_2_ storage, helps mitigate environmental liabilities from tailings disposal, and creates incentives for mining companies through potential carbon credit generation. Steel companies also produce large volumes of slag, some of which is recycled, while the rest is landfilled. Since slag contains minerals suitable for carbonation, it presents an opportunity for CO_2_ storage and waste reduction. The reported carbon capture capacity in the literature varies significantly, with amounts of 12–280 kg CO_2_ per tonne of EAF steel slags,^21,22^ and 5–183 kg CO_2_ per tonne of ultramafic mine materials.^23,24^ Several factors, including the mineralogy and composition of waste material, the chemical reagent used for leaching, and operating temperature and pressure, influence the rate of leaching and efficiency of carbonation. These factors may explain the discrepancies and variations reported in the literature.

In this work, we conducted a detailed investigation into CO_2_ mineralization using two types of industrial solid wastes (ISWs): Ca-rich EAF slag and Mg-rich mine tailings, under simulated flue gas conditions at atmospheric pressure. The process follows an indirect aqueous carbonation pathway involving two main stages: mineral dissolution/leaching and carbonate precipitation. To enhance leaching and carbonation efficiencies, key operational parameters—temperature, CO_2_ presaturation, and leaching agent selection—were systematically studied. A key innovation in this work is the investigation of ultrasound cavitation to enhance leaching and carbonation processes. This technique promotes chemical reactions by delivering localized high energy, breaking particles, improving solid–liquid contact, and enhancing mass transfer.^25–27^ Ultrasound-assisted leaching has shown significant improvements in metal recovery. For example, using ultrasound nearly doubled tungsten recovery from scheelite compared to nonsonicated systems.^28^ Liu et al.^29^ reported enhanced selective leaching of Ca and Mg from steel slag using ultrasound-assisted organic acid leaching. Kinetic analysis also revealed lower activation energies, indicating surface-controlled reactions. Similarly, Guo et al.^30^ found that ultrasound reduced the activation energy for nickel leaching from 17.74 to 5.04 kJ/mol.

Two acids were evaluated as leaching agents: a weak acid (AMD) and a strong acid (HCl), providing comparative insights into their effectiveness. AMD is a mining byproduct formed when sulfur-bearing minerals are exposed to air and water, resulting in acidic drainage that contains significant concentrations of dissolved Ca and Mg.^31^ In our previous work,^25^ we explored CO_2_ mineralization using raw AMD without solid feedstocks or additional extractants. The results demonstrated its potential as a viable strategy for the mining industry, offering both environmental remediation and revenue generation through carbon credits. Moreover, AMD’s acidity can aid in mineral leaching from solid feedstocks, enabling dual sourcing of minerals from both the solution and solids. To the best of our knowledge, only one study has investigated the use of AMD for treating simulated ultramafic tailings, with the existing literature primarily focused on proof-of-concept rather than operational implementation or optimization.^32^ HCl-based leaching, widely studied in CO_2_ mineralization of mineral wastes, offers high dissolution efficiency and selective extraction of reactive metals, making it a promising reagent for indirect carbonation processes.^33–39^ However, limited research has been conducted on the subsequent application of the resulting leachate in the CO_2_ carbonation stage.

Characterization techniques including XRF, ICP-OES, carbon analysis, TGA, and XRD were used to assess CO_2_ mineralization performance. These techniques helped identify optimal process configurations, advancing the mineralization approach as a competitive CCUS strategy.

## 2 Materials and Methods

### 2.1 Materials

Two solid waste feedstocks, obtained from the Canadian mining and steel industries, namely, nickel mine tailings and EAF steel slag, were utilized in this work. The acid solutions used were raw AMD, obtained from an active mine in Quebec, Canada, as well as 0.274 M HCl. Sodium hydroxide (NaOH) with 97% purity was used as an alkaline reagent. Ultrapure compressed CO_2_ and N_2_ gases (>99.99% purity) were used for the mineralization tests.

### 2.2 CO_2_ Mineralization Method

The setup consists of a gas supply system with mass flow controllers, a multiport reaction flask (1 L), a chemical metering pump, an ultrasound processor, a pH control unit, a water vapor condenser, and a CO_2_ gas IR analyzer. The detailed schematic of the CO_2_ mineralization test apparatus used can be found in the Supporting Information (Figure S1). For a typical mineralization experiment, the gas mixture composition was set at 12.5% CO_2_ using separate mass flow controllers for N_2_ and CO_2_. About 300 mL of acid solution was added to the reaction flask along with 3.75 g of solid feedstock, representing a solid-to-liquid ratio of 0.0125 g/mL. The experiments were carried out under two conditions, with and without CO_2_ presaturation, to assess the effect of pre-existing CO_2_ during the leaching step. Presaturation was achieved by bubbling the CO_2_ gas mixture into the slurry solution for 35 min using either a magnetic stir bar or an ultrasound processor. This duration was chosen based on initial tests to determine the approximate time required to saturate the solution at ambient temperature. While actual saturation time may vary, the same duration was used for every test to ensure consistency and comparability. After 35 min, the CO_2_ flow was stopped, and the solution continued mixing for an additional 30 min. In tests without CO_2_ presaturation, the solution was mixed for a total of 65 min.

Next, the CO_2_-containing gas mixture was introduced to the solution for 30 min while adjusting the solution pH to 9–10 using a 2 M NaOH reagent. A pH probe inserted in the reaction flask measured the pH throughout the experiment and was connected to a pH controller and dosing pump. The carbonation reaction speed is correlated to the concentration of carbonate ions; therefore, the selected pH range ensured a sufficiently rapid reaction. Once the 30 min carbonation period elapsed, the CO_2_ flow was stopped, and only N_2_ was left to flow into the reaction flask to purge the system. This continued until no CO_2_ was detected at the outlet by the CO_2_ analyzer, indicating the end of the carbonation reaction. The resulting slurry was then collected, filtered, and the solid product was dried at 60–65 °C prior to analysis. It is worth noting that liquid samples (approximately 5 mL) were taken using a syringe at the end of each stage of the process (i.e.,; first leaching with or without the presence of CO_2_ (35 min), second leaching without the presence of CO_2_ (30 min), carbonation (30 min), and N_2_ flushing).

The reaction acceleration study was performed using a 500W ultrasound generator, equipped with a 12.7 mm diameter probe (titanium alloy) and power control console. During the experiment, the amplitude of the ultrasound was set at 60% (pulse mode: 1 s ON and 2 s OFF). For tests without ultrasound, the reaction temperature was maintained within the desired range (ambient to 65 °C) using a heated oil bath and a programmable magnetic stirring hot plate, with the stirrer speed set at approximately 800 rpm. For ultrasound-assisted tests, the reaction was studied at ambient temperature to prevent overheating and overload of the ultrasound processor. The specific operating conditions for both leaching and carbonation experiments are given in Table 1.

**Table 1:** Operating Conditions for the Experiments

**Nickel Tailing Feedstock**						
**acid type**	**test no.**	**temp.; °C**	**CO**_**2**_ **conc.; vol %**	S/L ratio, g/mL	**CO**_**2**_ **preloading**	**ultrasound accelerator**
weak acid (AMD)	A-1	25–27	12.4	0.0125	yes	no
	A-2	25–27	12.4	0.0125	yes	yes
	A-3	25–27	12.4	0.0125	no	no
	A-4	25–27	12.4	0.0125	no	yes
	A-5	45	12.4	0.0125	yes	no
	A-6	65	12.4	0.0125	yes	no
strong acid (HCl)	A-7	25–27	12.4	0.0125	yes	no
	A-8	25–27	12.4	0.0125	yes	yes
	A-9	25–27	12.4	0.0125	no	no
	A-10	25–27	12.4	0.0125	no	yes
	A-11	45	12.4	0.0125	yes	no
	A-12	65	12.4	0.0125	yes	no
**EAF Slag Feedstock**						
weak acid (AMD)	B-1	25–27	12.4	0.0125	yes	no
	B-2	25–27	12.4	0.0125	yes	yes
	B-3	25–27	12.4	0.0125	no	no
	B-4	25–27	12.4	0.0125	no	yes
	B-5	65	12.4	0.0125	yes	no
strong acid (HCl)	B-6	25–27	12.4	0.0125	yes	no
	B-7	25–27	12.4	0.0125	yes	yes
	B-8	25–27	12.4	0.0125	no	no
	B-9	25–27	12.4	0.0125	no	yes
	B-10	45	12.4	0.0125	yes	no
	B-11	65	12.4	0.0125	yes	no

### 2.3 Characterization Methods

Metal concentrations in liquid samples were determined using inductively coupled plasma optical emission spectrometry (ICP-OES) analysis. For solid samples, 0.25 g of each sample was digested with 12 mL of aqua regia containing a 1:3 ratio of nitric acid and hydrochloric acid, heated at 105 °C for 1h, then cooled to room temperature. The digests were diluted to 50 mL with deionized water (DI) to produce a solution of 24% aqua regia that was then passed through a 0.45 μm filter. The filtered digests were subsequently diluted 40 times with 2% nitric acid for a final concentration of less than 30 mg/L. ICP-OES was performed using the Agilent 5900 Synchronous Vertical Dual View instrument, where the radio frequency (RF) power was set to 1.2 kW, the pump speed to 12 rpm, and the gas flows for the plasma and nebulizer were set to 13.5 L/min and 0.70 L/min, respectively. Analyses were performed using the ICP Expert V7.7.1 software (Agilent).

To characterize crystalline phases in solid products, powder XRD experiments were carried out on the Bruker D8 Advance instrument with Cu Kα (λ = 1.5418 Å) as the excitation radiation source. The instrument was operated at a voltage and current of 40 kV and 40 mA, respectively. The XRD patterns were collected at a range of 5–80° 2θ, with an increment of 0.02° 2θ and a scan speed of 1 s/increment. All XRD patterns were analyzed on the Diffrac.Eva V5.2 software (Bruker) and the crystallographic profiles were identified using the ICDD PDF 5+ 2025 database.

Wavelength Dispersive X-ray fluorescence (WDXRF) analysis was also conducted to measure the elemental distribution in the solid samples using a Rigaku Supermini 200 equipped with a three-position crystal changer with LiF (200) and PET crystals. It includes a 50 kV, 200 W Pd-anode X-ray tube, and utilizes a scintillation counter and F-PC detectors. 0.65 g of the sample was mixed with 10% lithium tetraborate and 5% LiNO_3_. The mixture was heated to 900–1050 °C using a Claisse TheOX Advanced fusion machine to form a homogeneous glass bead for the analysis. For calibration, standards with known concentrations of Al_2_O_3_, CaO, Fe_2_O_3_, K_2_O, MgO, MnO, Na_2_O, P_2_O_5_, SiO_2_, SO_3_, TiO_2_, and trace elements such as Sr, Zn, Cr, Zr, Ni, and Cu were used. Precision was ensured within specified tolerances, with a mass % error ranging from 0.009 to 0.33 for the major elements, and an error ranging from 12 to 31 ppm for the trace elements.

The elemental composition of the samples, specifically Carbon (C), Hydrogen (H), Nitrogen (N), and Sulfur (S), was determined using the Elementar Vario EL Cube instrument. Each sample was weighed to approximately 10–15 mg and compressed into tin boats to ensure complete combustion at 1115 °C. The analysis included three replicates per sample. Calibration was performed using the certified reference material Sulfanilamide from LECO, resulting in standard deviations of approximately 0.01% for nitrogen, 0.05% for carbon, 0.05% for hydrogen, and 0.17% for sulfur.

Thermogravimetric analysis (TGA) was conducted using the TGA Q5000 from TA Instruments. Each sample weighed approximately 15–25 mg and was placed in a platinum pan. The temperature was increased from room temperature to 950 °C at a rate of 5 °C per minute under nitrogen gas, with mass loss recorded continuously throughout the heating process. Temperature calibration of the analyzer was performed using five Curie point standards at 153, 358, 554, 745, and 931 °C. Weight calibration was conducted using a silver and brass weights.

The carbon content of a solid sample was corresponded to the amount of sequestrated CO_2_ in the solid carbonate using the following equation

1 $$m_{{CO}_{2}‐Seq}=\frac{C_{wf}\times m_{powder}\times {{\mathrm{CO}}_{2}}_{MW}}{C_{Mw}}$$

where, *m*_CO_2_–Seq_, C_wf_, C_MW_, CO_2_MW__, and *m*_powder_ are the weight of sequestrated CO_2_ as carbonates (g), carbon weight fraction in solid products from elemental analysis (C), carbon molecular weight (12.011 g/mol), CO_2_ molecular weight (44.01 g/mol), and weight of collected powder (g), respectively. The leaching efficiency of metal at the end of the leaching stage was also estimated using eq 2

2 $$\mathrm{metal}\;l\mathrm{eaching}\;\mathrm{efficiency}\;\left(\%\right)=\frac{{\mathrm{mt}}_{1}-{\mathrm{mt}}_{2}}{{\mathrm{mt}}_{1}}\times 100$$

where, *mt*_1_ and *mt*_2_ represent the weights of metal elements in initial and postleached solid samples, respectively. The value of *mt*_2_ was estimated based on the concentration of the metal in the postleaching liquid, as determined by ICP data. For the AMD solution, the concentrations of naturally existing Ca and Mg cations were excluded from the metal leaching efficiency calculations.

## 3 Results and Discussion

The AMD solution was analyzed through ICP-OES, with results shown in Table 2. The solid wastes were characterized using XRF and their metal compositions are given in Table 3.

**Table 2:** Metal Concentrations of Raw AMD from ICP-OES Analysis

	Ca	Mg	Al	Fe	Si	Mn	Cr	S	
element	(ppm)	(ppm)	(ppm)	(ppm)	(ppm)	(ppm)	(ppm)	(ppm)	pH
raw AMD	223.8–244.37	103.4–120.4	99.8–112.1	224.4–263.0	14.03–21.2	6.25–6.91	0.04–0.4	750.2–860.55	2.2–2.5

**Table 3:** Composition of Solid Feedstocks Obtained from XRF Analysis

	Ca	Mg	Al	Fe	Si	Mn	S	Ti	Ni	Cr
element	(wt %)	(wt %)	(wt %)	(wt %)	(wt %)	(wt %)	(wt %)	(wt %)	(ppm)	(ppm)
nickel tailings	0.37	25.7	0.19	3.27	16.9	0.09	0.24	0.02	1644	3641
EAF slag	25.71	5.47	2.82	14.57	7.2	0.05	0.08	0.86	12	5009

The mineralogical characteristics of the feedstocks and selected products from XRD analysis are presented in Figure 1. The nickel tailing feedstock mainly contains lizardite (Mg_3_Si_2_O_5_(OH)_4_), brucite (Mg­(OH)_2_), magnesioferrite (Fe_2_^3+^MgO_4_) and magnetite (Fe_3_O_4_) (Figure 1a). The EAF slag feedstock majorly comprises of brownmillerite (Ca_2_(Al,Fe^3+^)_2_O_5_), larnite (Ca_2_SiO_4_), magnetite (Fe_3_O_4_), and wustite (FeO) (Figure 1b).

**Figure 1:**
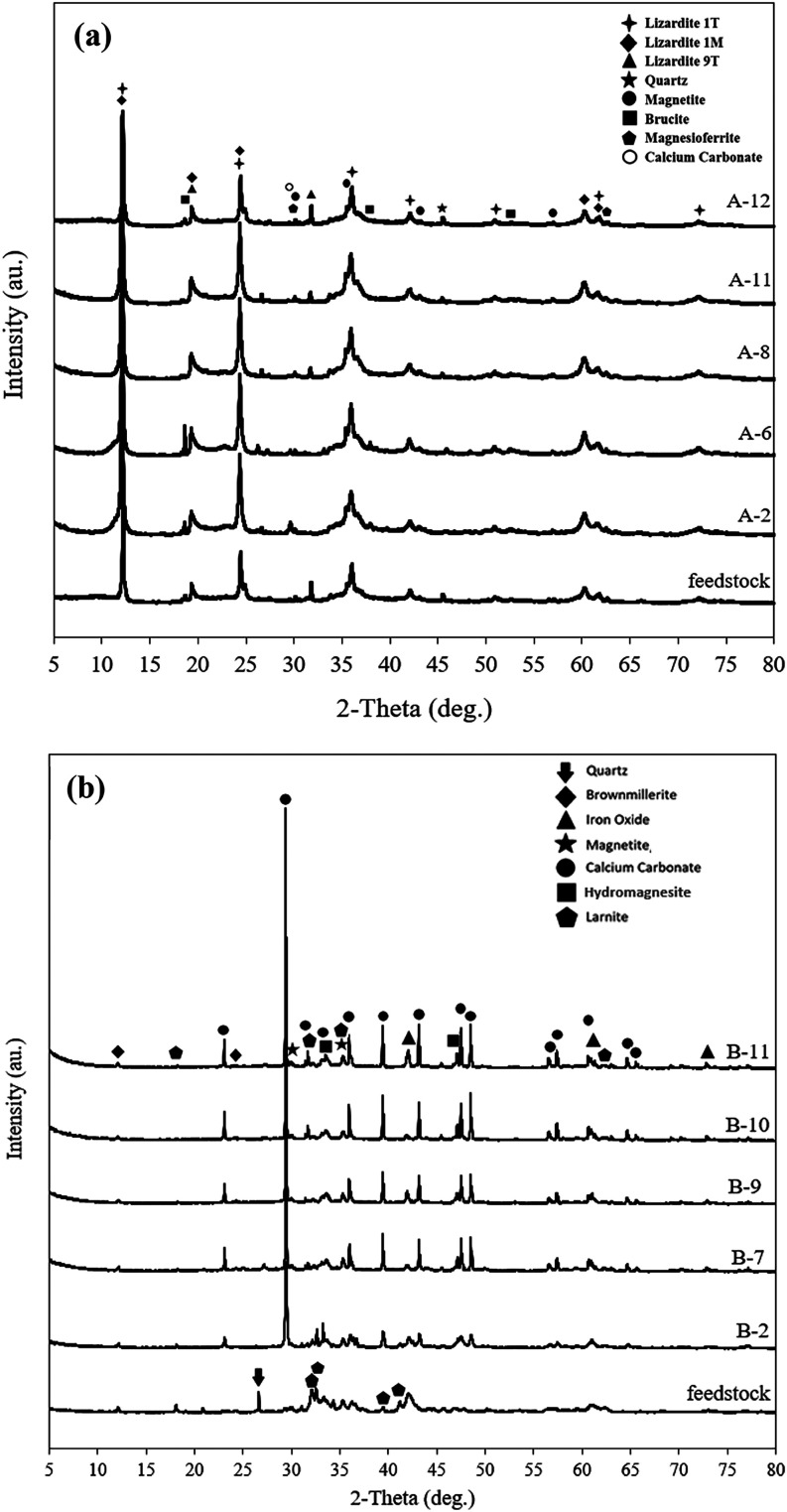
XRD of feedstocks and products; (a) nickel tailing, (b) EAF slag.

The results of the leaching and carbonation tests using the weak acid (AMD) and the strong acid (HCl) are presented in Figures 2, 3, and 4.

**Figure 2:**
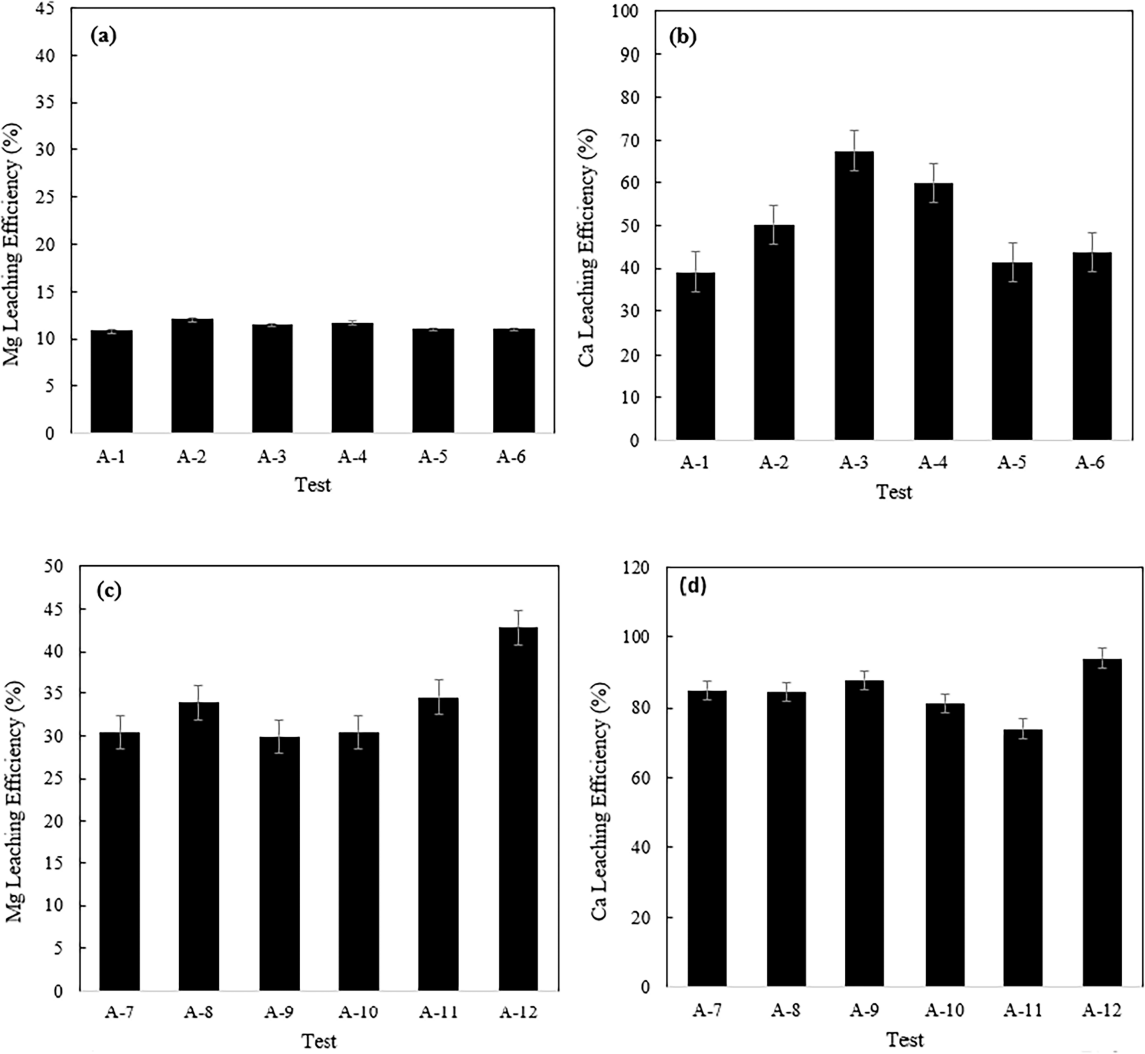
Mg and Ca leaching results for nickel tailing feedstock; (a) and (b): AMD solution, (c) and (d): HCl solution (the test details are provided in Table 1).

**Figure 3:**
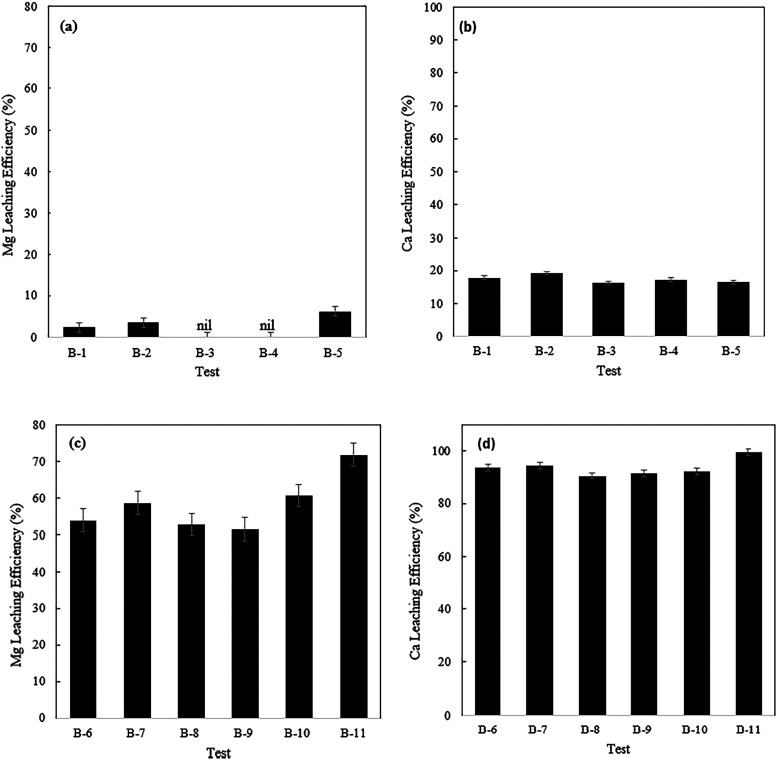
Mg and Ca leaching results for EAF slag feedstock; (a) and (b): AMD solution, (c) and (d): HCl solution (the test details are provided in Table 1).

**Figure 4:**
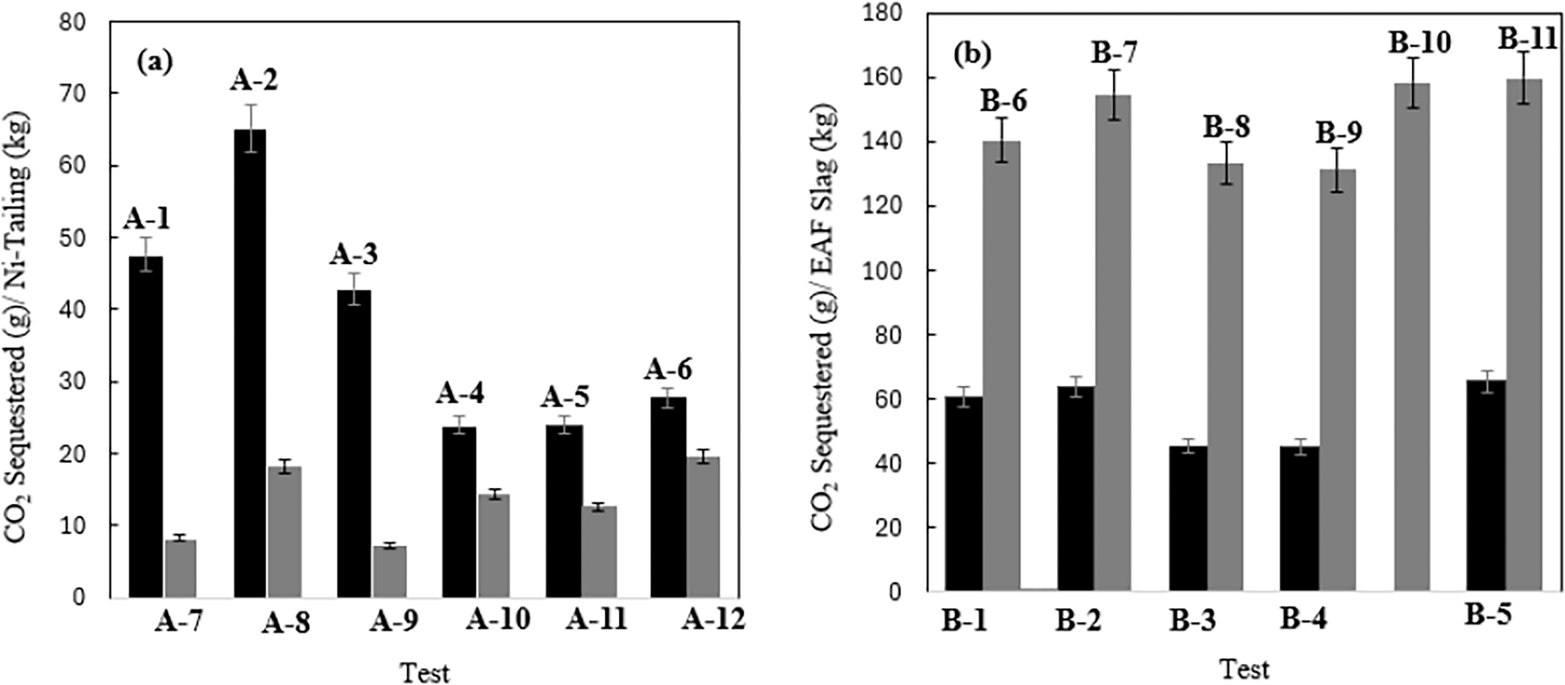
CO_2_ sequestration capacity of materials used; (a) nickel tailing, (b) EAF slag (tests A-1 to A-6 and B-1 to B-5 are with AMD; tests A-7 to A-12 and B-6 to B-11 are with HCl).

For nickel tailings, the effect of CO_2_ preloading on leaching was minimal, indicating that the presence of CO_2_ during leaching is not influential for Mg-rich minerals like serpentines. Additionally, a larger amount of CO_2_ sequestration was achieved with the weak acid. Nickel tailings are serpentine-based materials, known for their highly ordered crystalline structure, which makes them difficult to process with acid leaching chemicals.^12,40–43^

The leaching efficiencies of Ca and Mg with HCl were significantly higher than with AMD, due to the abundant availability of protons and stronger ionic strength, which facilitate the dissolution of metals into the solution. Additionally, the total amounts of Ca and Mg extracted into the solution were greater with HCl compared to AMD. Therefore, one might expect a higher carbonation capacity with HCl, which was not the case. There might be various reasons for observing such behavior. The raw AMD solution is richer in pre-existing dissolved Ca ions compared to HCl. Ca ions readily react with carbonate ions, making CaCO_3_ more likely to nucleate and grow than MgCO_3_ under varying solution conditions.^44^ Mg ions have a higher hydration energy than Ca ions. This means that Mg ions are more strongly bound to water molecules in aqueous solutions, making it more difficult for them to interact with carbonate ions and dehydrate to form MgCO_3_ during the carbonation process.^45–48^ Hence, metastable hydrated magnesium carbonates, such as nesquehonite or hydromagnesite, are more likely to form than magnesite, which is kinetically inhibited.^44,49^ Hydrated magnesium carbonates are more soluble and less stable compared to CaCO_3_, making their precipitation more difficult under ambient conditions. According to the literature, increasing the temperature can improve their precipitation by reducing their solubility and promoting their dehydration.^49–52^ Moreover, recent studies have shown that the presence of solution additives can influence the Mg ion dehydration step, as well as the early stages of MgCO_3_ nucleation and growth rates. These additives can shape the crystallization pathway of MgCO_3_ through either classical or nonclassical routes.^53–57^ Mg ions are highly hydrated in aqueous solutions, forming a stable coordination state of Mg­(H_2_O)_6_^2+^ in pure water.^58^ For carbonation nucleation to begin via binding with carbonate anions, a vacant site must be prepared by displacing water molecules. This leads to metastable under-coordinated configurations of hydrated Mg cations, a kinetically restricted route due to its high activation energy requirement.^52,58^ The energy requirement for promoting Mg cation dehydration can be decreased by adding solution additive anions such as fluoride, carboxylate, and bisulfide, which form contact cation–anion pairs and help stabilize under-coordinated configurations of hydrated Mg^2+^.^52,58^ Toroz et al.^57,58^ developed computational molecular modeling to simulate the hydration/dehydration mechanism of Mg cations in water, both in the absence and presence of several solution additive anions. According to their study, sulfate (SO_4_^2–^) tends to form contact ion pairs with Mg ions (Mg­(η^2^-SO_4_)­(H_2_O)_4_^2+^), which are thermodynamically more stable than the hexahydrated complex of Mg in water. Therefore, the presence of SO_4_^2–^ favors the dehydration state of Mg^2+^ and the nucleation of magnesium carbonate phases due to the formation of noncompetitive contact ion pairs. Conversely, Cl^–^ anions tend to form solvent-shared ion pairs with Mg ions, and the presence of Cl^–^ does not greatly interfere with the kinetics of Mg^2+^ dehydration, slightly improving the stability of the pentahydrated state of Mg ions. On the other hand, some research studies have investigated the coprecipitation of Mg and Ca ions and their interactions in solution.^59–64^ They noted that magnesium influences calcium precipitation, and some concluded that the coexistence of Mg ions promotes the formation of metastable aragonite instead of stable calcite.^59,65,66^ Additionally, some researchers reported that high amounts of Mg in seawater inhibit CaCO_3_ precipitation by decreasing calcite precipitation rates. However, this inhibition effect can be compensated by high pH, high salinity, or high temperature.^64,66^ The AMD solution used in this work had a low Mg/Ca molar ratio, and we did not observe any suppression of CaCO_3_ precipitation due to the presence of Mg ions.

Considering all the points mentioned, it can be concluded that the presence of sulfate ions in the AMD solution likely facilitated the precipitation of amorphous or poorly crystalline magnesium carbonate. Additionally, the presence of predissolved Ca in the AMD liquid provides an additional pathway for CO_2_ sequestration, beyond the carbonation of magnesium. This is further supported by the more pronounced CaCO_3_ peak (calcite: ∼29.43°) observed in Figure 1a, compared to the HCl-treated samples. This results in a higher CO_2_ sequestration capacity with AMD compared to HCl, which lacks sufficient Ca. This observation is also supported by TGA data for samples A-1 and A-2, presented in Figure 5. The initial weight losses at temperatures below 200 °C can be attributed to the dehydration of products such as hydrated magnesium carbonate or amorphous materials like Al­(OH)_3_ and Fe­(OH)_3_.^67–70^ Decarbonation of magnesium carbonate started at 250 °C, with the main weight loss occurring between 300 and 400 °C, primarily following the pattern for the decomposition of nesquehonite, hydromagnesite or Mg­(OH)_2_ as reported in the literature.^71–75^ Their weight loss spans a broader temperature range than that of the nickel tailings feedstock, whose Mg­(OH)_2_ decomposition occurs within a narrower range of 300–380 °C. The large weight loss between 450 and 750 °C is mainly due to the endothermic dehydroxylation of the lizardite mineral.^76–78^ As observed, the greater weight loss in tailing materials treated with AMD compared to HCl is likely due to the decomposition of magnesium and calcium carbonates, which aligns with their higher CO_2_ sequestration capacity. CaCO_3_ crystalline minerals are expected to primarily decompose within the temperature range of 450–850 °C,^79–81^ with calcite decomposition shifting downward for fine-grained particles, starting at temperatures as low as 600 °C.^82–84^ The small endothermic peak around 850 °C, detected for HCl-treated samples, can be attributed to the decomposition of crystalline coarse particles of calcite minerals.^85^

**Figure 5:**
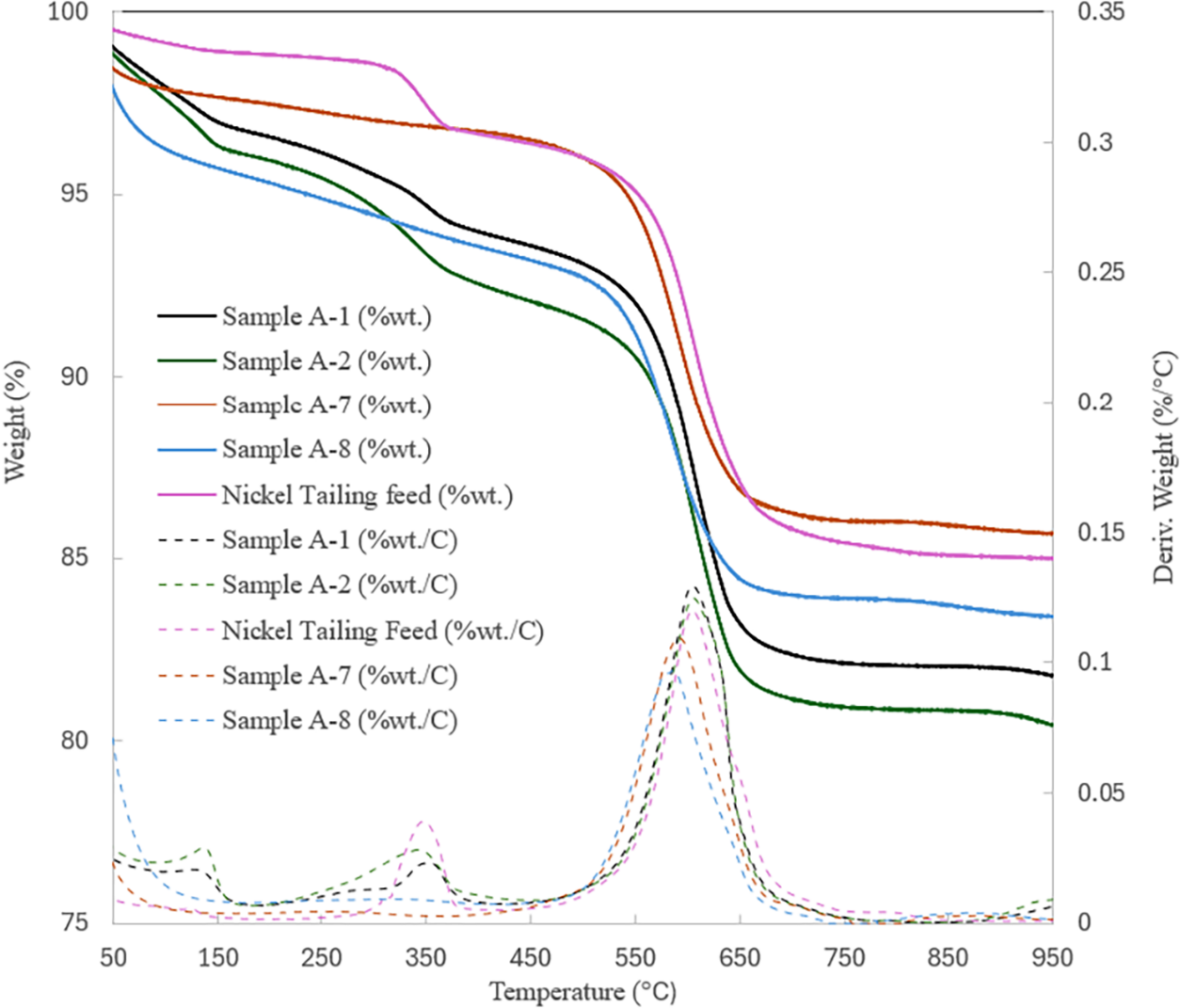
TGA data for carbonation products of nickel tailing sample, leached with AMD and HCl.

For ultrasound-assisted treatment, it is evident that Mg leaching efficiency improved. Consequently, higher Mg precipitation occurred at the end of the carbonation process, leading to a higher CO_2_ sequestration capacity compared to experiments without the ultrasound accelerator. Ultrasound processing enhances the leaching kinetics of metals by lowering the activation energy required for leaching reactions and increasing the frequency of molecular collisions.^86^ Additionally, the extreme local hot spots generated by ultrasound waves favor chemical reactions and enhance collisions and mass transfer. This also improves the diffusion of counterions (CO_3_^2–^) in solution, promoting the precipitation of Mg and Ca compared to processes without ultrasound.^25^ Two routes have been suggested for the crystallization of carbonate minerals: classical and nonclassical crystallization mechanisms.^53,87–90^ The classical route is generally initiated with the nucleation of unstable prenucleation clusters to form stable critical nuclei, followed with their further growth and crystallization.^89^ In the nonclassical mechanism, the process begins with the formation of thermodynamically stable prenucleation clusters. These clusters can grow and aggregate to form transitory amorphous intermediates or directly attach to growing crystal surfaces, eventually converting into the final crystalline phase.^52^ The benefits of ultrasound in crystallization arise from the combined effects of cavitation, mechanical, and thermal influences. Ultrasound enhances nucleation and growth rates, improves overall yield, and allows better control over product characteristics and morphologies.^91,92^ Xiang et al.^91^ reviewed the application of ultrasound in crystallization processes and noted that the crystallization of magnesium citrate was facilitated by ultrasonic microwave technology. Su et al.^93^ reported that ultrasound can increase the growth rate of calcium carbonate crystals in desalination processes. Stoica-Guzun et al.^94^ concluded that ultrasound processing influenced the size and morphology of calcium carbonate crystals, leading to a variety of shapes from calcite cubes to spherical and flower-like vaterite particles. Radfarnia et al.^25^ reached the same conclusion, finding that ultrasound-assisted neutralization of AMD solution advanced the crystallization of CaCO_3_. This resulted in reduced particle sizes and a mixture of rhombic calcite, spherical vaterite, and needle-like aragonite. During crystallization nucleation, solute molecules, being small and low-density, move close to the bubble walls, generated by cavitation, and gather there, forming prenucleation clusters. These clusters grow and eventually produce stable nuclei, which are released into the solution once they reach their critical sizes.^91^ The increase in nucleation rate in the presence of ultrasound can be attributed to the enhanced diffusion rate and collision frequency of solute molecules.^95^ Another benefit is that ultrasound processing can initiate secondary nucleation, which is triggered by pre-existing crystals.^96^ It can occur due to the erosion or wear of existing crystal nuclei, resulting in the formation of new nuclei and changes in crystal size.^97^

For the AMD solution, it was found that the Mg leaching efficiency remained almost unchanged with temperature variations. However, a specific trend could not be identified for Ca ions, likely due to solute concentration measurement errors associated with the small quantity of Ca in the feedstock and the marginal amount of leachable Ca (0.37 wt % of Ca vs 25.7 wt % of Mg in the feedstock). The leaching of Mg from serpentine via AMD appears to be primarily controlled by acid availability and mineral-specific dissolution kinetics rather than temperature within the studied range. AMD is a weak acid with limited H^+^ proton availability. As Mg dissolves, the protons are consumed, causing the solution pH to increase over time. At higher pH, the solution loses its leaching capability, and Mg leaching slows down regardless of temperature. On the other hand, the CO_2_ sequestration capacity showed a reduction. Increasing the temperature decreases CO_2_ solubility in water, reducing the availability of carbonate ions needed for magnesium and calcium precipitations. This shift may lead to the precipitation of magnesium hydroxide or mixed-metal hydroxides, reducing the net CO_2_ sequestration capacity per kg of feed. At higher temperatures, the solubility of Mg­(OH)_2_ decreases, causing it to precipitate more easily.^25,98^ This trend is evident in the current study, where a higher intensity of Mg­(OH)_2_ product was observed at an operating temperature of 60 °C compared to lower temperatures (Figure 1a). Figure 6 presents the pH changes of AMD and HCl solutions over time during the leaching step for nickel tailing.

**Figure 6:**
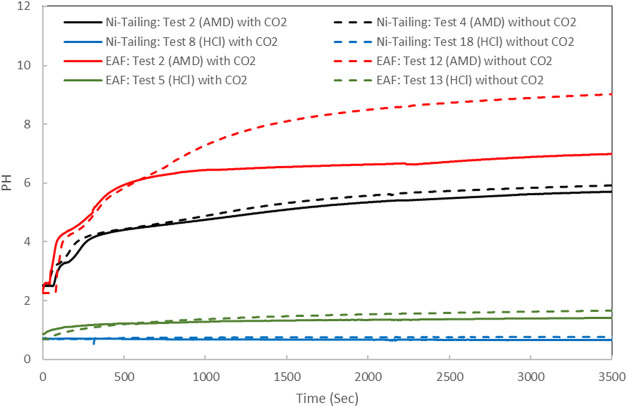
the pH changes over time during the leaching step for both EAF slag and nickel tailing.

Regarding the leaching of other main impurity metals, including Al and Fe, their concentrations in AMD solution were significantly reduced by the end of the leaching stage (Figure S2a,b). The rapid increase in pH after adding the tailing material (from 5.8 to 6.3) advanced their precipitation in the form of hydroxides or oxides.^99–102^ For the HCl solution, however, their concentrations mainly increased due to the favored leaching of metals in the presence of excess H^+^ in the solution. For the other major impurities, Si and S, their concentrations increased in both solutions, and no elemental precipitation was observed during the leaching stage (Figure S2c,d). With respect to the heavy metals, including Ni, Mn, Cr, and Ti, their leaching efficiencies were higher with the HCl solution compared to the AMD solution, as expected (Figure S2e,h). It appears that the concentrations of heavy metals did not decrease at the end of the leaching stage, as the pH increase was not sufficient for their precipitation. For Mn, the higher concentration achieved with AMD compared to HCl is attributed to the pre-existing Mn in the AMD solution. By the end of the carbonation stage, the concentrations of these elements in the solution were significantly lowered as a result of their hydroxide precipitation. For HCl solution, increasing the temperature improved Mg leaching efficiency, precipitation rate, and CO_2_ sequestration capacity (without ultrasound). Similar to AMD solution, ultrasound processing enhanced leaching and carbonation performance. The behavior of the CO_2_ preloaded solution was almost identical to the non- CO_2_ loaded one. Comparing leaching and carbonation results for tests with ultrasound at ambient temperature to those without ultrasound at higher temperatures (45 and 65 °C) revealed that a temperature increase was more influential than ultrasound for leaching. Unlike AMD, the CO_2_ uptake capacity was improved. This suggests that the carbonate formation rate may be influenced by the availability of magnesium ions in solution, which offsets the decrease in CO_2_ solvation capacity at elevated temperatures.

Although AMD is known as a weak acid, its CO_2_ sequestration capacity was found to be greater than that of HCl solution. One approach to boost CO_2_ sequestration capacity with HCl is to prepare the solution with a more Mg-supersaturated leachate for the carbonation reaction. This enhances the precipitation of magnesium carbonates, thereby increasing overall CO_2_ sequestration efficiency. The degree of supersaturation controls the precipitation rate, promoting reaction yield and consequently improving sequestration capacity.^49,50,103^ This can be achieved by applying a more concentrated HCl solution than the one used in this work during the leaching step. Studies have shown that the leaching performance of nickel tailings and serpentine-based ores is enhanced with increased HCl acid concentration.^11,104–106^ Choi et al.^104^ compared nickel tailing leaching with varied HCl concentrations from 0.5 to 2 M and concluded that Mg leaching efficiency increased from approximately 15 to 37% with higher acid concentrations. Hydrochloric acid, being a strong acid, fully dissociates in solution, producing hydronium ions. These ions break down the mineral structure of tailings and dissolve metals. Higher HCl concentrations increase hydronium ions, thereby improving leaching efficiency and metal leaching.

XRD analysis of post-treated nickel tailing samples (Figure 1a) shows that lizardite, the dominant mineral, remains largely unaffected by leaching, indicating its resistance to dissolution. The leaching of serpentine-based materials forms a passive layer of amorphous silica on the surface of serpentine minerals during dissolution. This layer acts as a barrier, significantly slowing down the leaching of magnesium by creating a protective coating that hinders access to the reactive sites underneath.^107–110^ For AMD-treated samples (A-2 and A-6), the increased intensity of the brucite peak at 18.61° indicates enhanced Mg­(OH)_2_ precipitation. This aligns with the high Mg^2+^ content in the AMD feed, which promoted brucite formation during carbonation, especially at elevated temperatures. On the other hand, for HCl-treated sample, it seems that the brucite was partially dissolved, supported by the corresponding peak intensity decrease. This suggests that AMD and HCl acid primarily extract the magnesium from the simply structured magnesium-bearing minerals like brucite or magnesioferrite, not lizardite polymorphs. While no crystalline magnesium carbonate was detected by XRD, the increase in total carbon content and TGA weight loss patterns consistent with decomposition of hydrated magnesium carbonates (nesquehonite or hydromagnesite) suggest the possible formation of poorly crystalline or amorphous magnesium carbonate phases. Their small amounts in the samples compared to the dominant residual tailings matrix, such as lizardite, may also obscure low-intensity signals of minor carbonate phases. Regarding EAF slag experiments, CO_2_ preloaded solutions showed greater CO_2_ sequestration capacity for both AMD and HCl applications. This contrasts with the results for nickel tailing, indicating that the presence of CO_2_ is more influential for leaching Ca-rich minerals. When CO_2_ is introduced during the leaching step, it dissolves in water and partially dissociates, releasing protons (H^+^) and bicarbonate ions. The solubility of CO_2_ in the solvent is generally limited and depends on temperature, pressure, and solute concentrations.^111^ EAF slag, being highly alkaline, raises the solution pH to the alkaline range when hydrolyzed in water.^112^ According to Le Chatelier’s Principle, the consumption of protons by reacting with hydroxyls favors CO_2_ dissolution and carbonic acid dissociation. This promotes the hydrolysis of alkaline minerals and the leaching of metals such as Ca and Mg. For AMD solution, the combination of alkalinity from EAF slag hydrolysis and acidity from carbonic acid dissociation buffers the solution pH to around 7.0 by the end of the leaching stage. At this point, most of the equilibrium CO_2_ exists as bicarbonate anions. Notably, without CO_2_ preloading, the solution pH reached over 9.0, likely leading to the precipitation of Ca­(OH)_2_ and Mg­(OH)_2_.^98^ A portion of the Mg or Ca cations could be confined in an amorphous precipitate of Mg­(OH)_2_ or Ca­(OH)_2_, as their crystalline phases were not detected in the corresponding XRD graphs (Figure 1b). This may also help explain the lower CO_2_ sequestration capacity observed without CO_2_ preloading. From Figure 6, the different pH trends in AMD solution during the leaching stage with EAF slag is obvious, depending on whether CO_2_ is present or absent. In contrast to the nickel tailing trend, where the buffering effect of CO_2_ preloading was negligible, the presence of CO_2_ during leaching had a notable impact on EAF slag processing. This difference may explain the significant effect of CO_2_ in EAF slag leaching processing. For HCl solution, the abundance of hydronium ions and higher ionic strength likely reduce CO_2_ solubility,^113^ and the dissociation of carbonic acid is expected to be smaller compared to AMD. Nevertheless, the CO_2_ buffering effect is still evident for EAF slag treated with HCl solution, unlike nickel tailing, when compared to tests without CO_2_ preloading (Figure 6). This could slightly improve metal leaching performance. Due to the low pH value in HCl solutions (pH < 2.0), the majority of the equilibrium CO_2_ in the bulk liquid is expected to remain in a free state at the end of the leaching stage. Similar to the nickel tailing study, the leaching efficiencies of Ca and Mg metals using HCl were significantly higher compared to AMD. This is due to the higher ionic strength of HCl, which facilitates the dissolution of metals into the solution. Stronger acids enhance metal mineral leaching by providing more H^+^ ions, breaking down the mineral structure and accelerating dissolution. The higher leaching of Mg and Ca using EAF slag compared to nickel tailing is due to Ca-bearing minerals like larnite (Ca_2_SiO_4_) being readily leached by acids without forming a dense silica-rich passivation layer. In contrast, lizardite minerals form such layers, hindering magnesium leaching. Based on Figure 1b, it can be observed that the peaks associated with the larnite, the main Ca-bearing constituent, has been weakened. This confirms that calcium silicate contributed to the leaching process. Comparable to nickel tailing, the concentrations of other primary impurity metals, i.e., Al and Fe in the AMD solution were significantly reduced by the end of the leaching stage, due to the precipitation of their hydroxides or oxides (Figure S3a,b). For the other impurities, including Si and heavy metals Ni, Cr, Mn, and Ti, the metal ion concentrations leaching into the solution were significantly higher with HCl compared to AMD (Figure S3d,h). At the end of the carbonation stage, their concentrations in the solution were dramatically reduced due to the formation of their hydroxides. The CO_2_ sequestration capacity of the HCl leached solution was more than twice than that of the AMD processed EAF slag. The solution from HCl treatment is much richer in Ca and Mg compared to the AMD solution, where these ions readily precipitate out when the pH is adjusted above 9.0. Increasing the temperature to 65 °C improved the CO_2_ sequestration capacity of the EAF slag samples for both AMD and HCl, particularly enhancing Mg leaching. The slightly lower Ca leaching for AMD may be due to measurement errors or the formation of gypsum in the sulfate-containing solution, which reduces overall Ca leaching efficiency. Gypsum can form in calcium-sulfate bearing solutions at temperatures as low as 60 °C.^25,114^ Our ICP-OES data (Figure S3c) indicated a greater decrease in sulfur concentration at the end of the leaching stage at 65 °C compared to ambient conditions (683.9 ppm vs 729.9 ppm). Although no crystalline gypsum was detected in XRD patterns (Figure 1b, B11), its amorphous phase may have formed. The increase in CO_2_ sequestration capacity with temperature can be attributed to an improved carbonate precipitation rate. The carbonation of Ca and Mg is temperature-dependent.^50,115–118^ Therefore, the increased rate of carbonate precipitation in Ca and Mg enriched solutions likely outweighs the decrease in CO_2_ solubility in the solution. The CO_2_ sequestration capacities and metal leaching efficiencies with ultrasound were also higher, as observed in the nickel tailing processing, due to improved mass transfer, diffusion, and nucleation rates.

TGA data for some EAF slag samples are presented in Figure 7. As shown, the weight loss corresponding to the amount of CO_2_ sequestered using the HCl-treated sample is much larger than that for the AMD-processed solution. Additionally, the TGA results indicate a significantly larger amount of CaCO_3_ produced in samples presaturated with CO_2_, as evidenced by Tests B-2 and B-7 compared to Tests B-4 and B-9. This confirms that presaturation with CO_2_ enhances CaCO_3_ formation. The peaks observed at temperatures below 200 °C are attributed to the dehydration of products, including hydrated magnesium carbonate as well as amorphous Al­(OH)_3_ and Fe­(OH)_3_. For HCl-treated samples, the primary weight loss can be observed over a wider temperature range of 250–700 °C compared to the samples treated with AMD. For both treatments, the weight loss observed in the range of 300–400 °C can be attributed to the thermal decomposition of nesquehonite or hydromagnesite, as previously discussed with nickel tailing materials. For HCl-treated samples, two primary endothermic peaks are evident at 550–600 and 660–680 °C. In contrast, AMD-treated samples exhibit a single main endothermic peak at 650–660 °C, primarily due to the decomposition of fine-grained calcite. As shown in Figure 1b, crystalline calcite is the main product of carbonation. The weight loss associated with the first primary peak for HCl-treated samples is attributed to the loss of hydroxyl groups resulting from the decomposition of amorphous Fe­(OH)_3_ and/or Al­(OH)_3_, or partial decomposition of amorphous CaCO_3_, if present. The second primary peak is mainly related to the thermal decomposition of calcite. Fe­(OH)_3_ typically dehydroxylates between 400–500 °C, resulting in the formation of FeO­(OH).^119^ Higher endothermic peaks (550–600 °C) observed here can be associated with the transition of FeO­(OH) to other crystalline phases such as hematite.^67,120^ Similarly, according to literature, thermal decomposition of amorphous Al­(OH)_3_ to AlOOH and γ-Al_2_O_3_ and/or α-Al_2_O_3_ is complete by 700 °C.^70,121^ HCl solvent is more selective for Fe and Al compared to AMD solvent (Figure S3a,b), allowing a larger amount to be leached out from EAF slag material. These elements are then precipitated as their amorphous hydroxides during the carbonation stage, which are detectable during TGA analysis. Additionally, a peak at around 850 °C for the B-7 and B-9 samples (treated with HCl) can be associated with the release of CO_2_ from the well crystalline larger particles of calcite.

**Figure 7:**
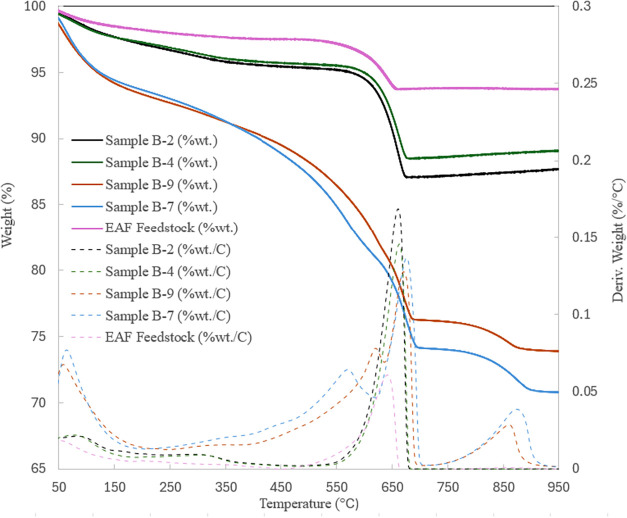
TGA data for carbonation products of EAF slag sample, leached with AMD and HCl.

The overall cost of mineralization processes is influenced by various factors, including feedstock characteristics, process conditions, energy consumption, chemical reagents, and potential revenue streams. Feedstock pretreatment requirements, such as grinding and heat activation, along with reaction conditions like pressure and temperature, are determining factors.^122^ High-pressure and high-temperature carbonation processes typically accelerate reaction kinetics but also increase energy demands. Optimizing these parameters is crucial for balancing process efficiency and cost-effectiveness. Additionally, the selection and consumption of chemical reagents, such as acids for leaching and alkaline agents for carbonation, contribute to operating costs. Using recycled reagents or industrial waste streams can help mitigate costs. Using AMD as a leaching agent offers some economic advantages by providing a cost-effective alternative to acids such as HCl, reducing the need for chemical purchases. Moreover, utilizing AMD helps mitigate environmental liabilities associated with its treatment and disposal, potentially lowering remediation costs for mining operations. However, the effectiveness of AMD as a leaching agent is highly dependent on the characteristics and minerology of the waste feedstock used, which can influence the efficiency of metal leaching and subsequent carbonation. On another note, as shown in this work, integrating ultrasound into the leaching process of steel slag and nickel tailing wastes enhanced the performance of their leaching and carbonation processes. However, it is important to consider the associated energy consumption and costs. The overall energy efficiency depends on specific process parameters and the material being treated. The energy required for ultrasound-assisted leaching can vary based on factors such as ultrasonic power, frequency, mode of operation, and duration. For instance, Kobus et al.^123^ reported that pulsed ultrasound modes can reduce electricity consumption compared to continuous modes in certain leaching processes. With a gross power consumption of 500 W by the ultrasound processor used in this work, the energy consumption can be estimated at approximately 0.54 kWh for the leaching stage and 0.25 kWh for the carbonation stage. From Schlömer et al.,^124^ this translates to average CO_2_ equivalent emissions of approximately 649.2, 387.9, 182.1, 38, 8.7, and 19 g when the electricity is sourced from pulverized coal-fired, natural gas combined-cycle, biomass power plants, solar, wind onshore, and hydro power plants, respectively. Considering the CO_2_ uptake of 65.1 g-CO_2_/kg-waste for nickel tailings and 154.8 g-CO_2_/kg-waste for EAF slag achieved using the ultrasound technique, the ultrasound application results in a negative CO_2_ emissions intensity, if the electricity is sourced from renewable energies. It should be noted that the current study did not focus on optimizing the conditions, and the CO_2_ emission intensity can certainly be decreased by optimizing influencing parameters such as duration, amplitude, pulsation characteristics, etc. While ultrasound equipment may involve additional capital costs, these can be offset by benefits such as reduced processing times, increased metal recovery rates, and lower reagent consumption. Nonetheless, a comprehensive cost-benefit analysis and further study to optimize the operation mode is essential to determine the economic viability of implementing ultrasound-assisted leaching in steel slag and nickel tailing treatment.

## 4 Conclusions

This study investigated CO_2_ mineralization using alkaline industrial wastes, specifically electric arc furnace (EAF) steel slag and nickel mine tailings. By utilizing both HCl and waste-derived AMD leaching agents, the research assessed metal leaching efficiency and CO_2_ sequestration potential under varying conditions, including temperature and ultrasound-assisted acceleration. The results showed that AMD could serve as a cost-effective alternative to HCl, with its effectiveness dependent on the mineralogical composition and type of the feedstocks. For magnesium-rich nickel tailings, AMD achieved higher sequestration capacity than HCl, while for calcium-rich EAF steel slag, HCl was more effective. Moreover, increasing temperature or using ultrasound processing improved CO_2_ sequestration efficiency. These findings underscore potential for developing sustainable strategies for industrial waste valorization and greenhouse gas mitigation.

## Supplementary Material



## Notes

Figure S1. Detailed schematic of CO2 mineralization test setup. Figure S2. Nickel tailing metal ion concentrations from ICP-OES; (a) Fe, (b) Al, (c) S, (d) Si, (e) Ni, (f) Mn, (g) Cr, (h) Ti. Figure S3. EAF slag metal ion concentrations from ICP-OES; (a) Fe, (b) Al, (c) S, (d) Si, (e) Ni, (f) Mn, (g) Cr, (h) Ti (PDF)
