# Supplementary material for: Experimental Study on Indirect CO2 Mineralization of Industrial Solid Wastes: Electric Arc Furnace (EAF) Slag and Nickel Mine Tailings
Source: ACS Omega. 2025 Sep 4;10(36):41571–85. doi: 10.1021/acsomega.5c05128 (PMC12444509; doi:10.1021/acsomega.5c05128)
Supplement: Supplementary file 1 [file ao5c05128_si_001.pdf]

SUPPORTING INFORMATION

Experimental Study on Indirect CO_2 Mineralization of Industrial Solid Wastes: Electric Arc Furnace (EAF) Slag and Nickel Mine Tailings

Hamid Radfarnia*[1], Katrin Staneva[1], Ahmed Shafeen[1], Kourosh Zanganeh[1], Bussaraporn Patarachao[2], Stephannie Vasquez Huertas[2], Andre Zborowski[2], Judy Kung[2], Seyedeh Laleh Dashtban Kenari[3], Sanaz Mosadeghsedghi[3], Konstantin Volchek[3]

[1]Natural Resources Canada, CanmetENERGY, 1 Haanel Drive, Ottawa, ON K1A 1M1, Canada

[2] National Research Council, Energy, Mining and Environment, 1200 Montreal Road, Ottawa K1A 0R6, Canada

[3]Natural Resources Canada, CanmetMINING, 555 Booth Street, Ottawa, ON K1A 0E9, Canada

* Corresponding author

E-mail addresses: hamid.radfarnia@nrcan-rncan.gc.ca (Hamid Radfarnia)

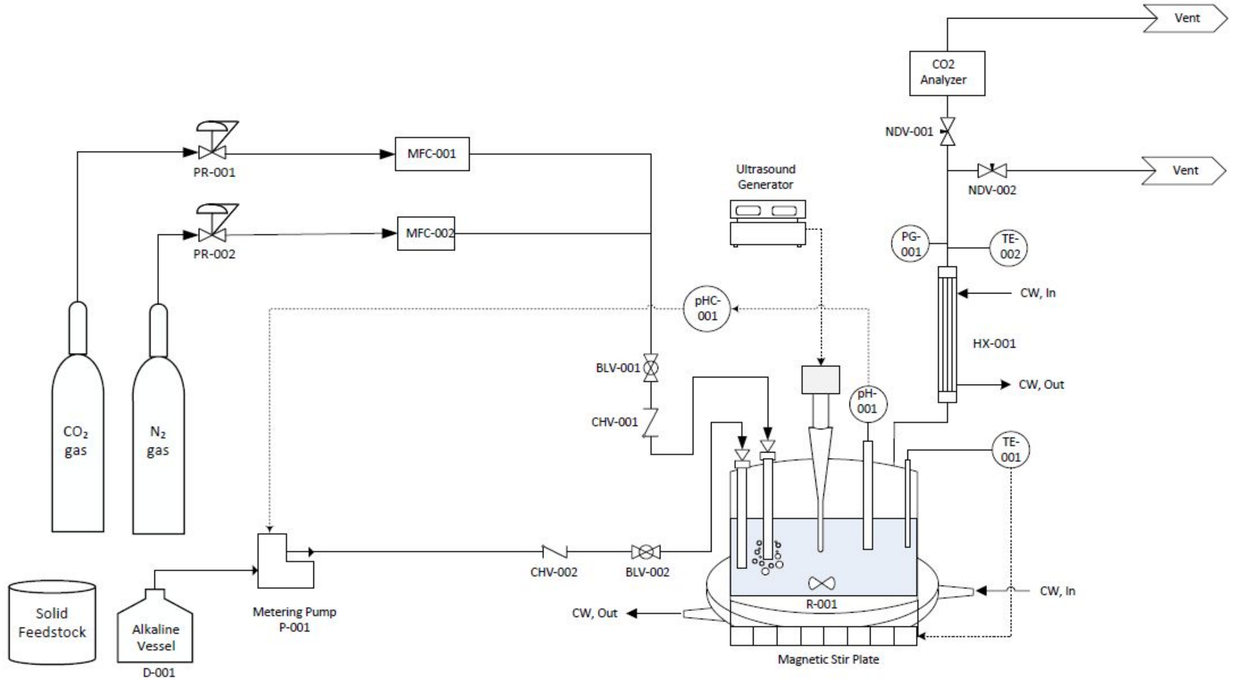

Figure S1. Detailed schematic of CO_2 mineralization test set-up.

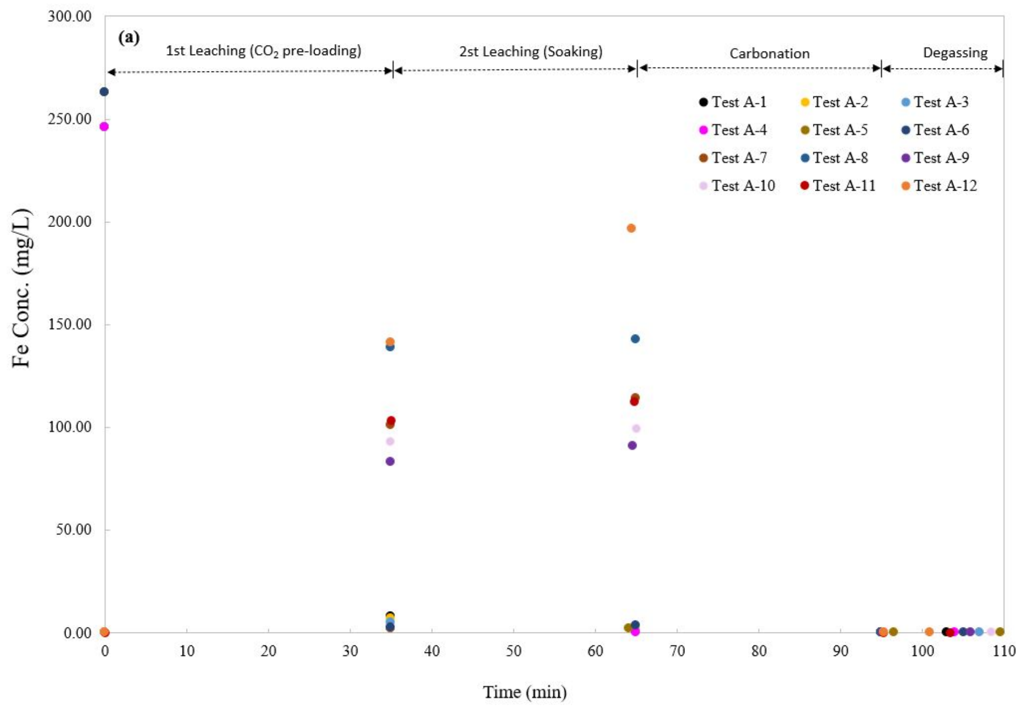
(a)
1st Leaching (CO2 pre-loading)
2st Leaching (Soaking)
Carbonation
Degassing
Test A-1
Test A-2
Test A-3
Test A-4
Test A-5
Test A-6
Test A-7
Test A-8
Test A-9
Test A-10
Test A-11
Test A-12
Fe Conc. (mg/L)
Time (min)

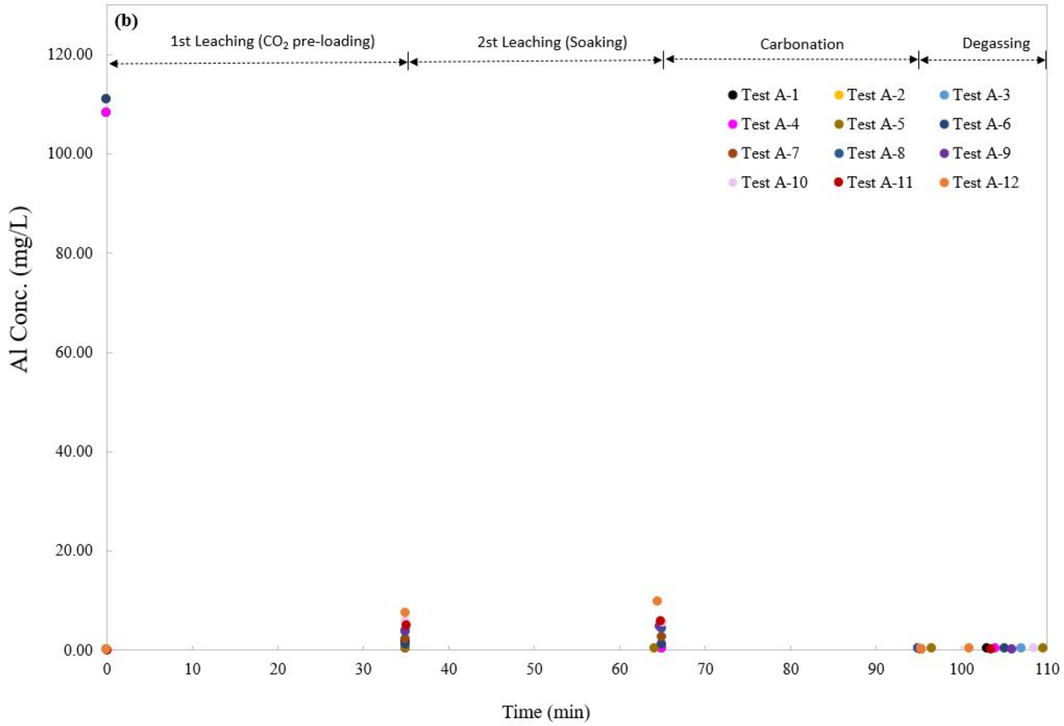
(b)
1st Leaching (CO2 pre-loading)
2st Leaching (Soaking)
Carbonation
Degassing
Test A-1
Test A-2
Test A-3
Test A-4
Test A-5
Test A-6
Test A-7
Test A-8
Test A-9
Test A-10
Test A-11
Test A-12
Al Conc. (mg/L)
Time (min)

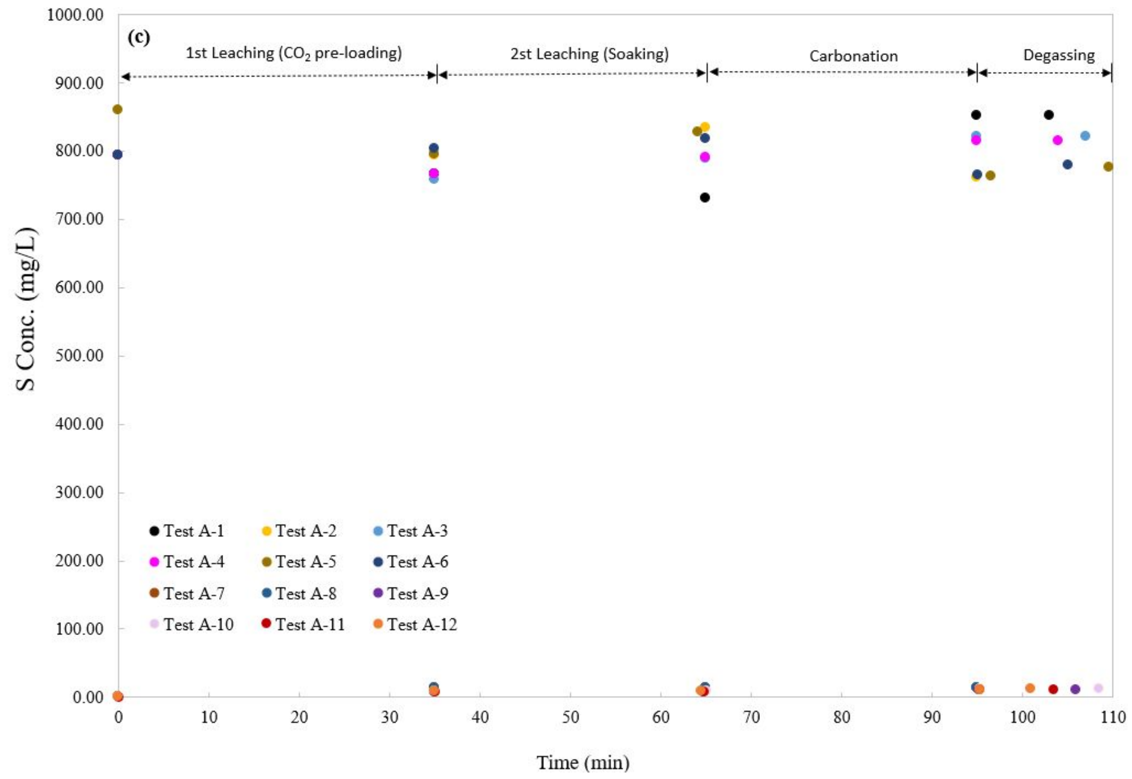
(c)
1st Leaching (CO2 pre-loading)
2st Leaching (Soaking)
Carbonation
Degassing
S Conc. (mg/L)
Time (min)
Test A-1
Test A-2
Test A-3
Test A-4
Test A-5
Test A-6
Test A-7
Test A-8
Test A-9
Test A-10
Test A-11
Test A-12

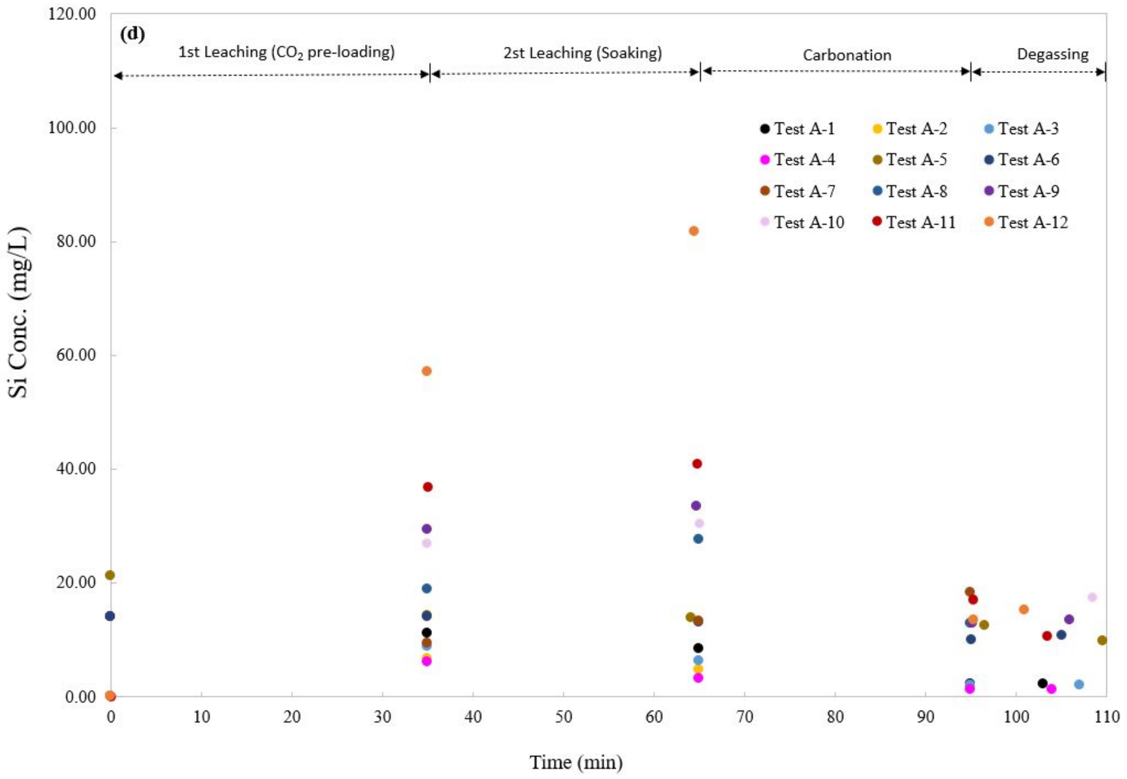
(d)
1st Leaching (CO2 pre-loading)
2st Leaching (Soaking)
Carbonation
Degassing
Si Conc. (mg/L)
Time (min)
Test A-1
Test A-2
Test A-3
Test A-4
Test A-5
Test A-6
Test A-7
Test A-8
Test A-9
Test A-10
Test A-11
Test A-12

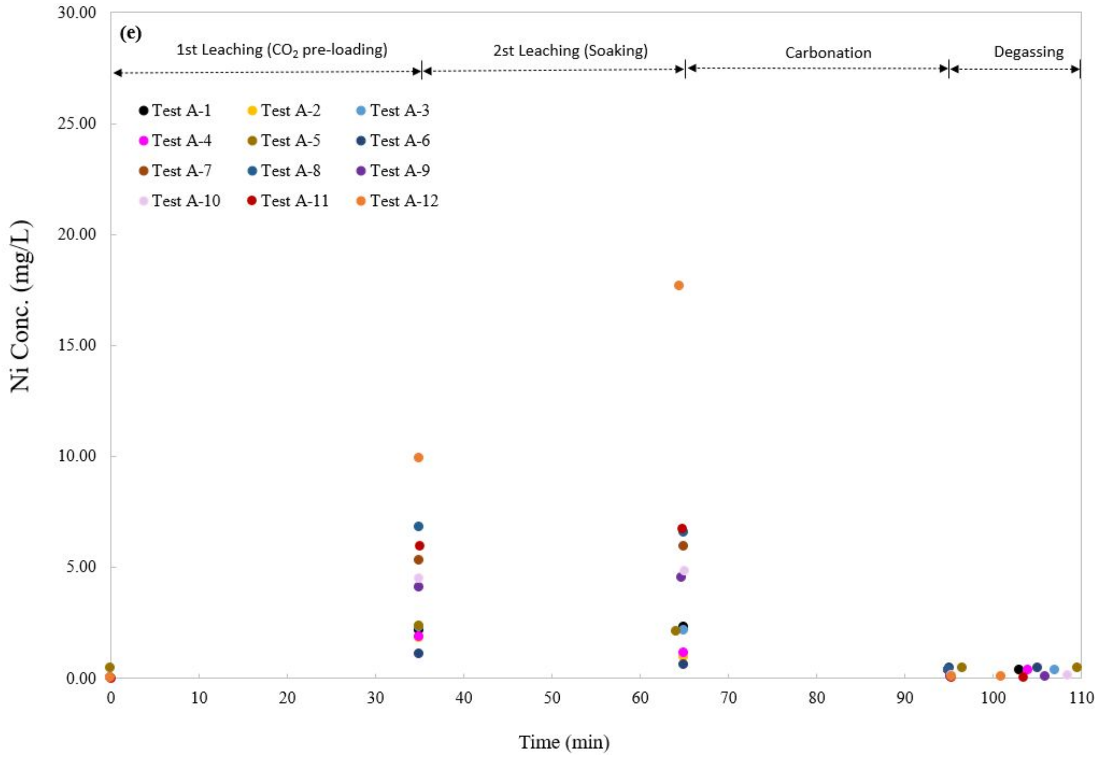
(e)
1st Leaching (CO2 pre-loading)
2st Leaching (Soaking)
Carbonation
Degassing
Test A-1
Test A-2
Test A-3
Test A-4
Test A-5
Test A-6
Test A-7
Test A-8
Test A-9
Test A-10
Test A-11
Test A-12
Ni Conc. (mg/L)
Time (min)

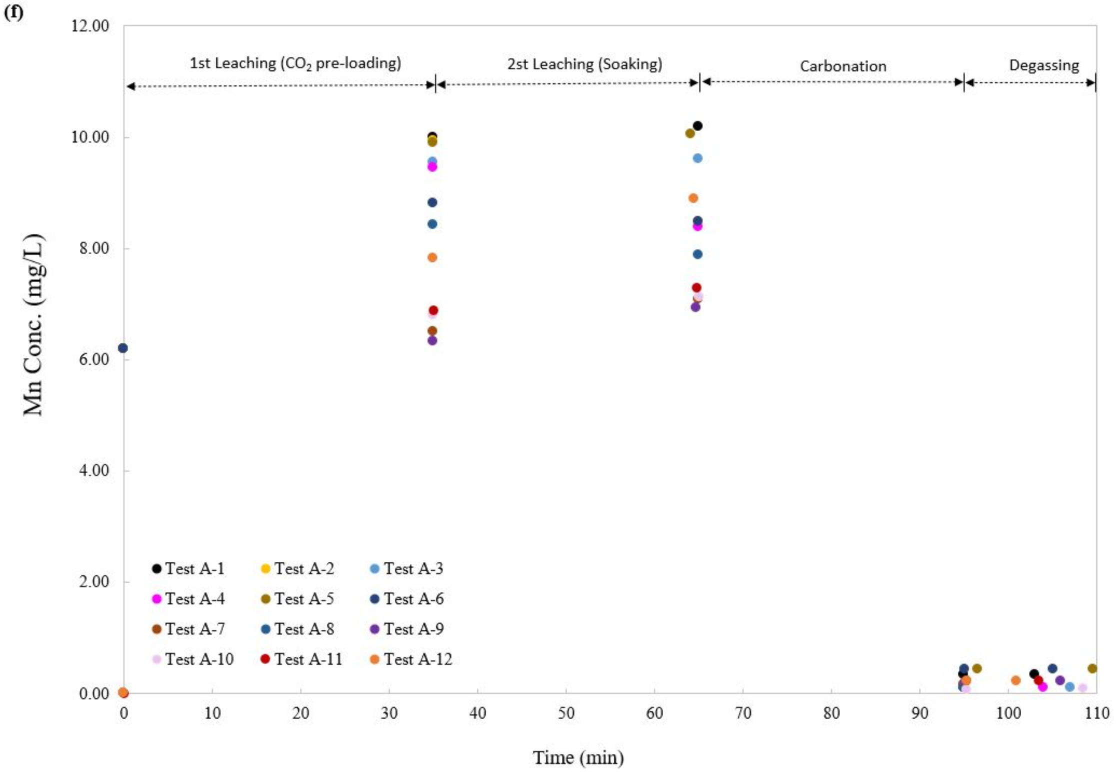
(f)
1st Leaching (CO2 pre-loading)
2st Leaching (Soaking)
Carbonation
Degassing
Test A-1
Test A-2
Test A-3
Test A-4
Test A-5
Test A-6
Test A-7
Test A-8
Test A-9
Test A-10
Test A-11
Test A-12
Mn Conc. (mg/L)
Time (min)

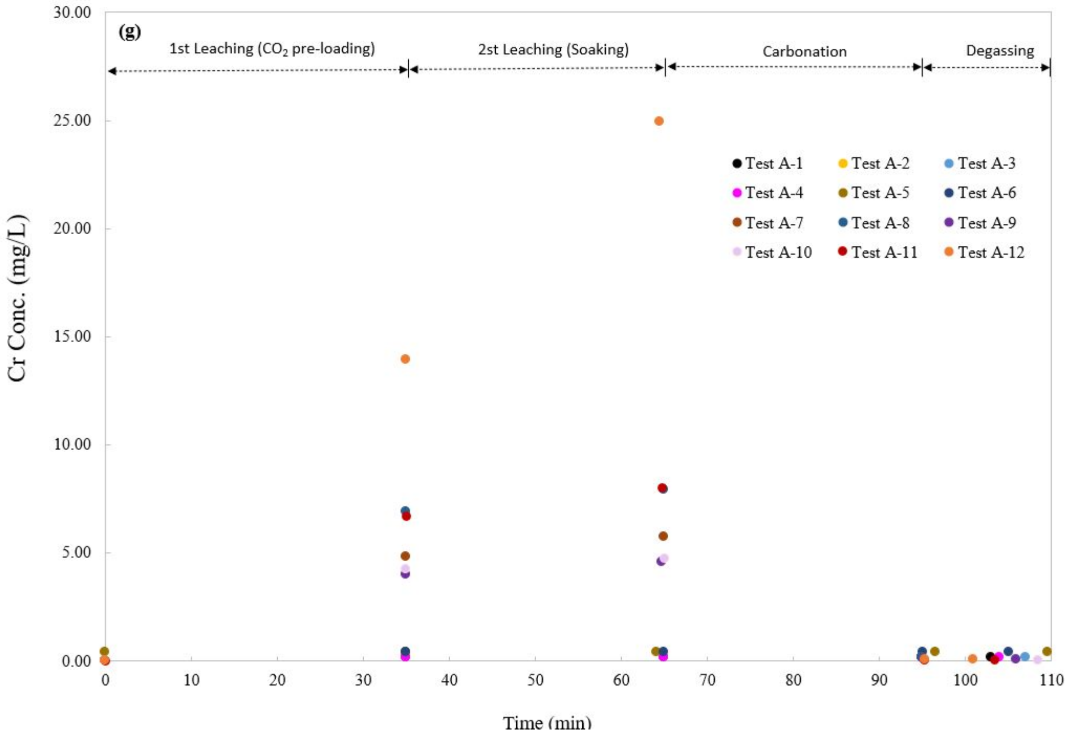

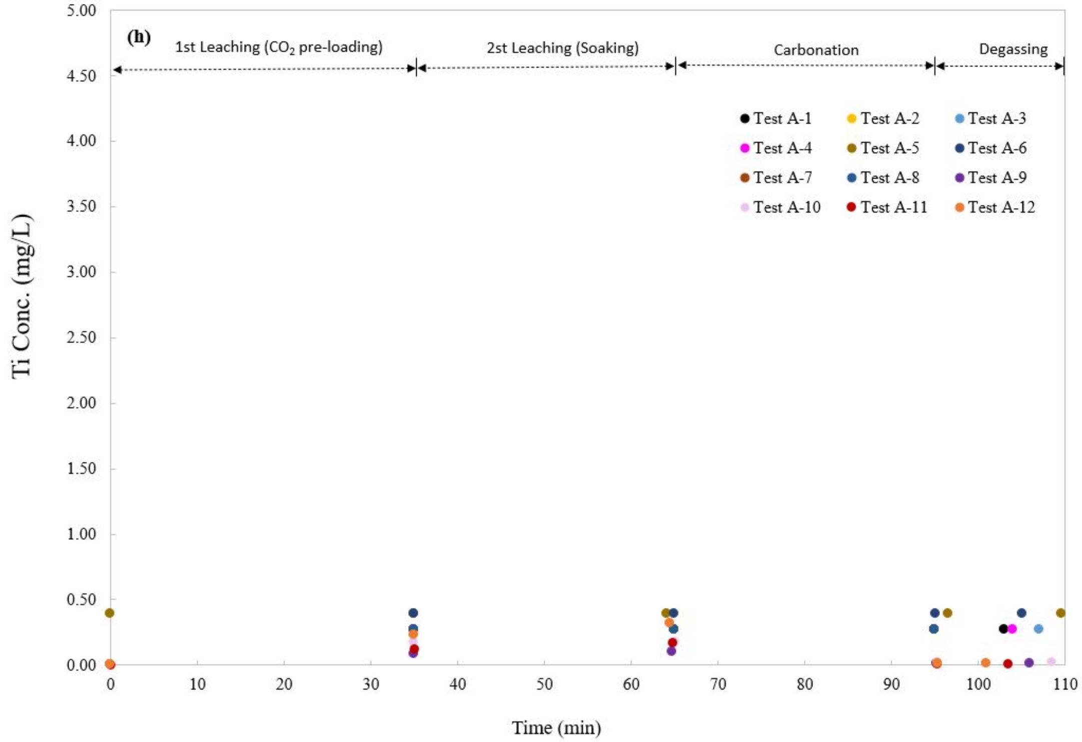

Figure S2. Nickel tailing metal ion concentrations from ICP-OES; (a) Fe, (b) Al, (c) S, (d) Si, (e) Ni, (f) Mn, (g) Cr, (h) Ti.

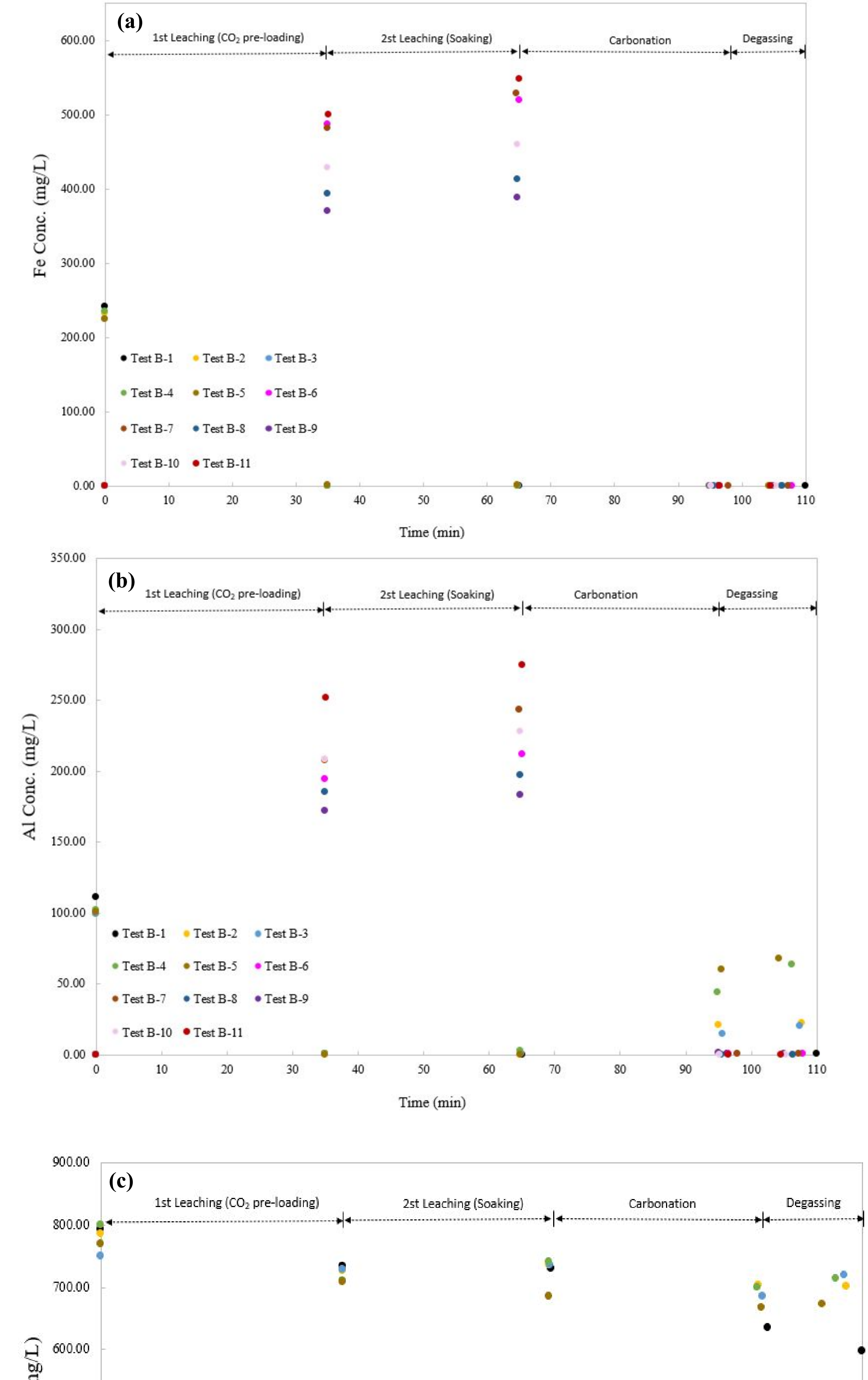
(a)
1st Leaching (CO2 pre-loading)
2st Leaching (Soaking)
Carbonation
Degassing
Fe Conc. (mg/L)
Time (min)
Test B-1
Test B-2
Test B-3
Test B-4
Test B-5
Test B-6
Test B-7
Test B-8
Test B-9
Test B-10
Test B-11
(b)
1st Leaching (CO2 pre-loading)
2st Leaching (Soaking)
Carbonation
Degassing
Al Conc. (mg/L)
Time (min)
Test B-1
Test B-2
Test B-3
Test B-4
Test B-5
Test B-6
Test B-7
Test B-8
Test B-9
Test B-10
Test B-11
(c)
1st Leaching (CO2 pre-loading)
2st Leaching (Soaking)
Carbonation
Degassing

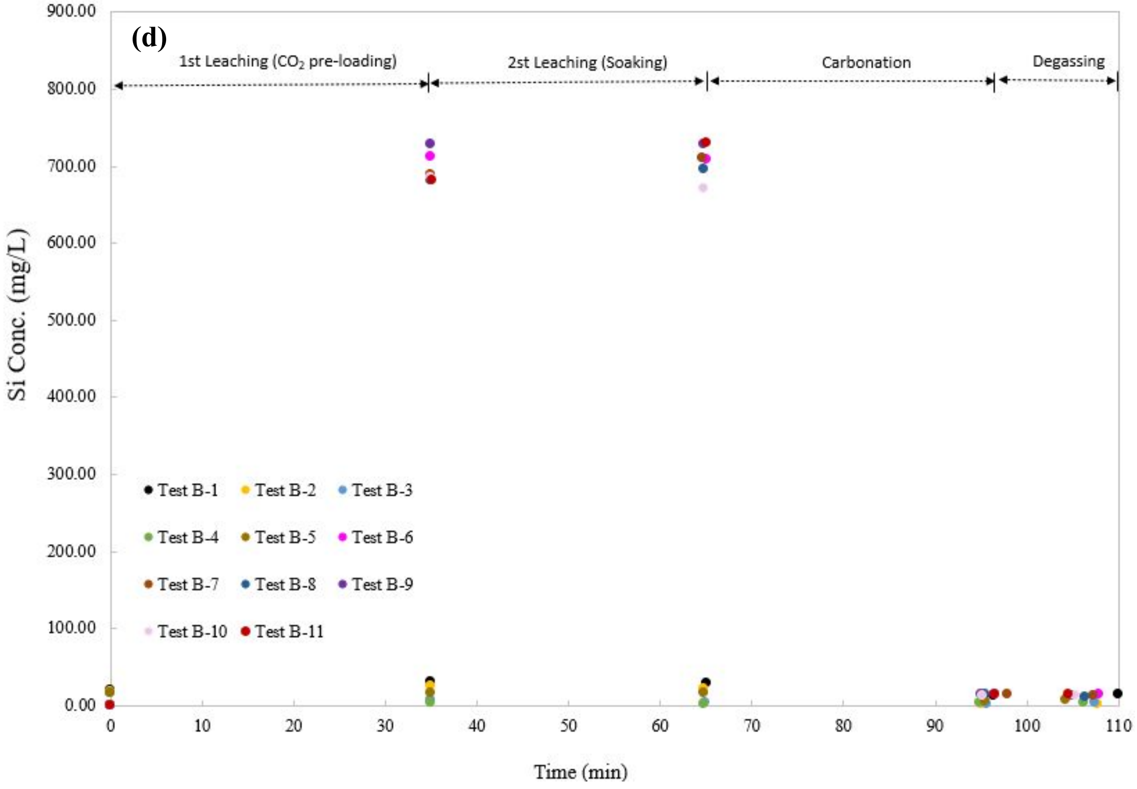

(d)
1st Leaching (CO2 pre-loading)
2st Leaching (Soaking)
Carbonation
Degassing
Si Conc. (mg/L)
Time (min)
Test B-1
Test B-2
Test B-3
Test B-4
Test B-5
Test B-6
Test B-7
Test B-8
Test B-9
Test B-10
Test B-11

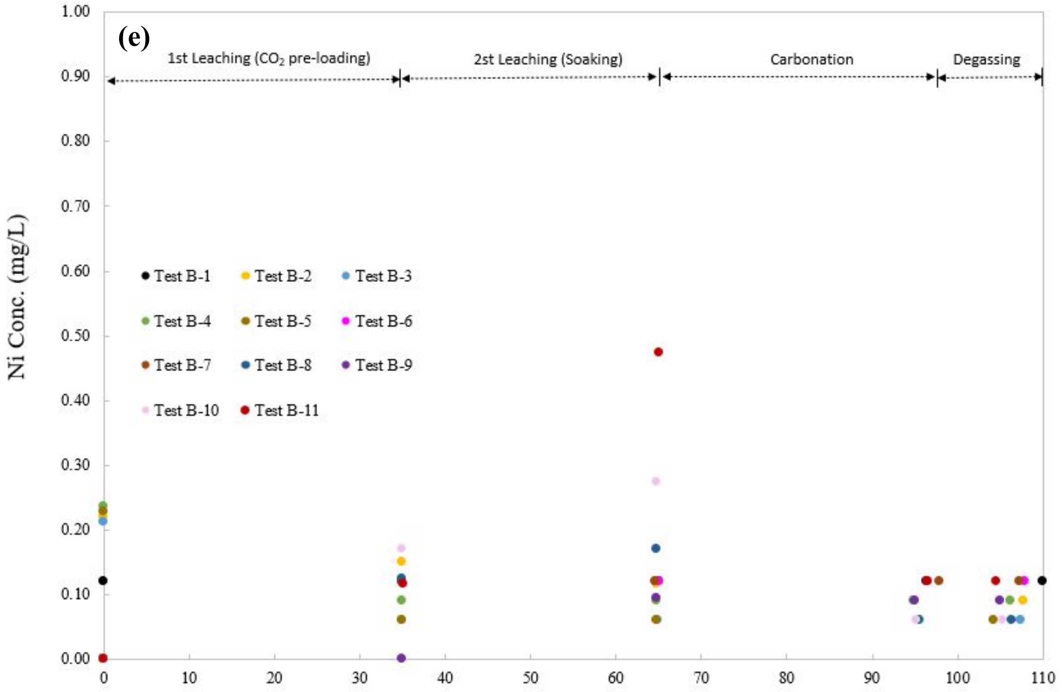
(e)
1st Leaching (CO2 pre-loading)
2st Leaching (Soaking)
Carbonation
Degassing
Ni Conc. (mg/L)
Test B-1
Test B-2
Test B-3
Test B-4
Test B-5
Test B-6
Test B-7
Test B-8
Test B-9
Test B-10
Test B-11
1.00
0.90
0.80
0.70
0.60
0.50
0.40
0.30
0.20
0.10
0.00
0
10
20
30
40
50
60
70
80
90
100
110

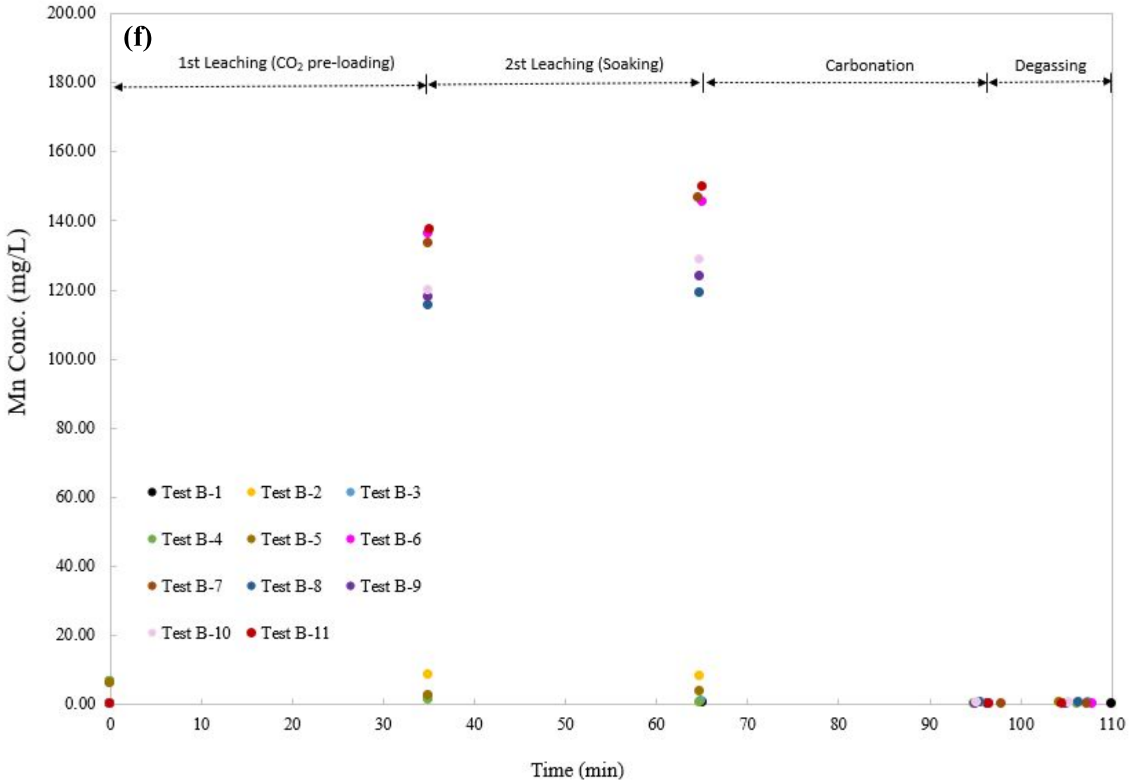
(f)
1st Leaching (CO2 pre-loading)
2st Leaching (Soaking)
Carbonation
Degassing
Mn Conc. (mg/L)
Test B-1
Test B-2
Test B-3
Test B-4
Test B-5
Test B-6
Test B-7
Test B-8
Test B-9
Test B-10
Test B-11
200.00
180.00
160.00
140.00
120.00
100.00
80.00
60.00
40.00
20.00
0.00
0
10
20
30
40
50
60
70
80
90
100
110
Time (min)

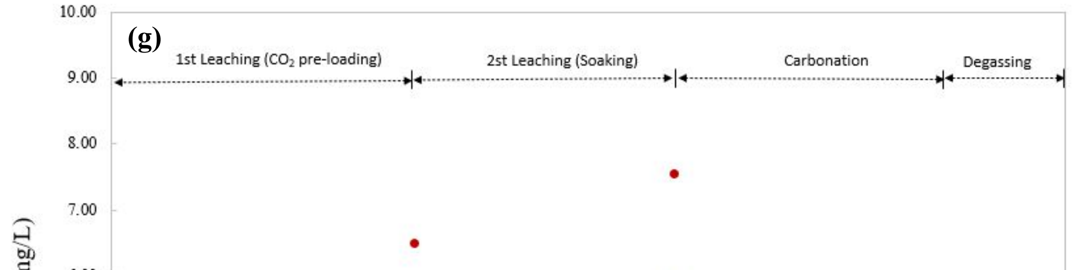
(g)
1st Leaching (CO2 pre-loading)
2st Leaching (Soaking)
Carbonation
Degassing
10.00
9.00
8.00
7.00

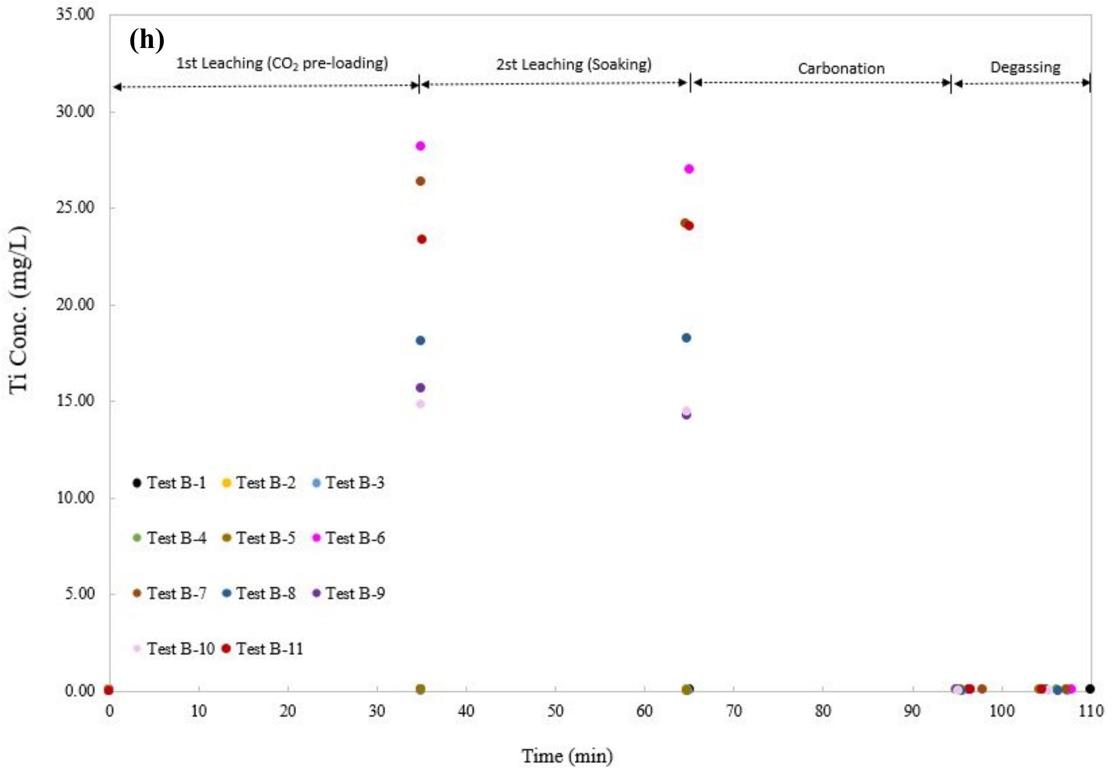

Figure S3. EAF slag metal ion concentrations from ICP-OES; (a) Fe, (b) Al, (c) S, (d) Si, (e) Ni, (f) Mn, (g) Cr, (h) Ti.
